# Burden of mental health and substance use disorders among Italian young people aged 10–24 years: results from the Global Burden of Disease 2019 Study

**DOI:** 10.1007/s00127-022-02222-0

**Published:** 2022-01-20

**Authors:** Simone Amendola

**Affiliations:** Department of Dynamic and Clinical Psychology, Faculty of Medicine and Psychology, Sapienza University of Rome, Via degli Apuli 1, 00185 Rome, Italy

**Keywords:** Mental health, Burden of disease, Trends, Youth, Italy

## Abstract

**Purpose:**

The burden of mental health and substance use disorders among Italian young people have not yet been presented in detail, despite adolescents and young adults aged between 10 and 24 years constitute 14.5% of the Italian population. Therefore, the aim of this study was to provide data on the health burden of mental health and substance use disorders among young people (10–24 years) in Italy between 1990 and 2019.

**Methods:**

Ecological study design using data from the Global Burden of Disease Study 2019. Age- and sex-specific prevalence and years lived with disability (YLDs) of mental health and substance use disorders with the uncertainty intervals were reported as well as their percentual changes between 1990 and 2019.

**Results:**

Prevalence and YLDs rates of mental health and substance use disorders showed negative trends overall between 1990 and 2019. However, diagnoses of attention-deficit/hyperactivity, autism spectrum, conduct and eating (among males) disorders increased as well as cocaine use disorder. The highest levels of disability in terms of YLDs were due to anxiety, depressive, conduct and eating disorders and alcohol use, amphetamine use and opioid use disorders. The disease burden was higher in middle-late adolescence and young adulthood than early adolescence, among females than males for mental health disorders and among males compared to females for substance use disorders.

**Conclusion:**

Findings of the study highlighted disorder-specific patterns of prevalence and YLDs rates and were discussed considering previous research. The public health system should continuously sustain mental health promotion and prevention efforts in young people.

**Supplementary Information:**

The online version contains supplementary material available at 10.1007/s00127-022-02222-0.

## Introduction

Adolescence is the life phase in which future patterns of adult psychophysical health are established [1]. Accordingly, public health systems need to focus their attention on the promotion of mental and physical health of young people. Ballester et al. [2] explored the age of onset of mental health disorders among 2118 Spanish university students. The authors showed that the age of onset of adult attention hyperactivity, mood and anxiety disorders, alcohol and drug abuse/dependence was between 14 and 19 years. Similarly, Cía et al. [3] reported that the median age of onset of any disorder was 20 years of age among 3927 Argentinean adults. Furthermore, the median age of onset of post-traumatic stress, anxiety, mood and substance use disorders varied between 15 and 21 years among a sample of 671 soldiers of the United States [4]. These findings indicate that the onset of several psychiatric disorders is more common during the adolescent years and, consequently, young people mental health is central to many health agenda and policy [1].

Causes of health loss and mortality among children and adolescents substantially improved in many countries between 1990 and 2017 [5]. The decreases in disease burden due to infectious, neonatal and nutrition associated causes have been followed by the increasing role of injuries and noncommunicable diseases, such as mental health and substance use disorders [6]. In particular, mental health and substance use disorders were the seventh leading cause of disability-adjusted life years in low- and middle-income countries in 2010, while they ranked first in high-income countries [7]. Furthermore, depression, anxiety and conduct disorders increased in global ranking from 1990 to 2015 for people aged 19 years or younger [6].

A recent study analysed the burden of mental health disorders among children and adolescents aged 5–14 years in Europe observing that mental health disorders in 2015 ranked second among the causes of years of life lived with disability (YLDs) [8]. Specifically, conduct disorders, anxiety disorders, autism–Asperger syndrome and depressive disorders were among the twenty diseases that caused the most health burden in 2015 in Europe. At the same time, children and adolescents’ mental health improved between 2000 and 2015.

Gore et al. [9] described for the first time the global burden of disease and the associated risk factors in young people aged 10–24 years using data from the 2004 Global Burden of Disease (GBD) study. Their findings indicated that neuropsychiatric disorders were the main cause of burden in high-income countries. Depressive disorders, schizophrenia, bipolar and alcohol use disorders were among the top ten causes of health burden. Moreover, self-harm was the first most common cause of injury related death among adolescents in many European countries in 2013, indicating the importance of early detection and effective management of mental health and substance use disorders as part of suicide prevention strategies [10].

The continuous monitoring of the mental health of young people is essential to maintain and consolidate the progress made in the last decades as well as to understand where interventions are having an impact [5, 10]. Moreover, levels and trends of the leading causes of death and disability among the young people are crucial to guide investment and inform policies as emphasized by the Global Burden of Disease Paediatrics Collaboration [10]. According to a public and global mental health perspective, targeting sensitive periods of development by investing in the mental health and well-being of young people as well as in quality research plays an essential role in the promotion of mental health [11].

Adolescents and young adults aged between 10 and 24 years constituted 14.5% of the Italian population in 2019 [12]. However, the data on mental health and substance use disorders among Italian young people have not yet been presented in detail, despite previous research reported findings of GBD studies in Europe. Italy has faced a time of economic and demographic decline characterized by a decrease in the fertility rate and an increase in life expectancy resulting in fewer children and adolescents, but more middle-aged and older people [13, 14]. At the same time, the high unemployment rate among Italian young adults constitutes a matter of concern [15, 16]. Taken together, these aspects have implications for the future sustainability of effective health care and pension systems [17]. Consequently, even more attention should be paid on the mental health of young people.

In light of the above, the aim of the present study was to describe and analyse the health burden of mental health and substance use disorders among Italian adolescents and young adults aged 10–24 years using data from the GBD 2019.

## Methods

### The Global Burden of Disease study

The estimates of mental health and substance use disorders for Italian young people aged 10–24 years were extracted from GBD 2019. The GBD 2019 study produced consistent estimates of 369 of diseases and injuries for 204 countries and territories, including Italy. The methods of the GBD 2019 study have been previously described [18]. All GBD estimates are publicly available and adhere to the Guidelines on Accurate and Transparent Health Estimate Reporting [19].

### GBD cause hierarchy, measures and analysis presented

Mental health disorders are defined on the basis of the clinical diagnostic criteria from the Diagnostic and Statistical Manual of Mental Disorders (fourth edition, text revision) or the International Classification of Diseases (tenth edition) [20].

In the GBD 2019 cause hierarchy, mental health and substance use disorders are on Level 2, under noncommunicable diseases (Level 1). Mental health disorders are divided into schizophrenia, depressive, bipolar, anxiety, eating, autism spectrum, attention-deficit/hyperactivity and conduct disorders, idiopathic developmental intellectual disability and other mental disorders (Level 3). Depressive disorders are subdivided to major depressive disorder and dysthymia, while eating disorders to anorexia and bulimia nervosa (Level 4), however, the data on these subcategories were not considered for analysis. Furthermore, the data on conduct disorders are reported only for the age groups 10–14 and 15–19 years.

Substance use disorders are divided into alcohol and drug use disorders (Level 3). Drug use disorders are subdivided into opioid, cocaine, amphetamine, cannabis and other drug use disorders (Level 4). The data on opioid and amphetamine use disorders are presented only for the age groups 15–19 and 20–24 years.

The data on Italy from 1990 to 2019 are presented, including prevalence and YLDs. YLDs are years lived with disability (in which the disability equates to a fraction of a year lived in full health) and are the product of the mean duration of the condition in years and the disability weight of that condition [14, 20].

The analysed and presented data include count estimates of prevalence and YLDs, age-specific prevalence and YLDs rates per 100,000 population, percentage changes from 1990 to 2019 for prevalence and YLD rates both total and by sex among young people aged 10–24 years. Furthermore, age-specific prevalence and YLDs rate per 100,000 people in 2019 are present by age groups (10–14, 15–19 and 20–24 years). All estimates were accompanied by uncertainty interval (95% UI). The data visualization tools, from which the data were extracted, are publicly available online (http://ghdx.healthdata.org/gbd-results-tool).

## Results

### Mental health disorders in 2019 in people aged 10–24 years

In 2019, there were 1.4 (95% UI 1.3–1.6) millions of young people suffering from mental health disorders in Italy with a rate of 16637 (95% UI 14880–18701) per 100,000 young people (Online Resource 1). In the same year, 186.9 (95% UI 131.2–255.9) thousand of YLDs were attributed to mental health disorders among Italian young people with a rate of 2144 (95% UI 1506–2937) per 100,000 young people.

Anxiety, depressive and attention-deficit/hyperactivity disorders were the three most common mental health disorders diagnosed among young people in 2019 (Online Resource 1). In Italy, 0.6 (95% UI 0.5–0.8), 0.3 (95% UI 0.2–0.3) and 0.2 (95% UI 0.1–0.3) millions of young people suffered from anxiety, depression and attention-deficit/hyperactivity disorders, respectively, with a rate of 7052 (95% UI 5538–8881), 3093 (95% UI 2414–3884) and 2408 (95% UI 1676–3414) per 100,000 young people. Moreover, anxiety, depressive and conduct disorders were the three diagnoses associated with the highest burden in terms of YLDs in 2019. In particular, 60.5 (95% UI 40.7–86.4), 50 (95% UI 31.9–74.6) and 21.6 (95% UI 12.2–34) thousand of YLDs were attributed to anxiety, depressive and conduct disorders, respectively, among Italian young people in 2019 with rates of 694.6 (95% UI 466.7–992.1), 574.4 (95% UI 365.8–856.6) and 248.4 (95% UI 139.7–390.3) per 100,000 young people.

### Substance use disorders in 2019 in people aged 10–24 years

0.3 (95% UI 0.2–0.4) million of Italian young people were diagnosed with substance use disorders overall in 2019, with a rate of 3195 (95% UI 2487–4072) per 100,000 young people (Online Resource 2). The YLDs attributed to substance use disorders were 29.8 (95% UI 19.8–41.8) thousand with a rate of 341.5 (95% UI 227.1–480) per 100,000 young people.

Alcohol use, cannabis use and amphetamine use disorders were the three most common substance use disorders diagnosed among young people in 2019 (Online Resource 2). In Italy, 132.5 (95% UI 90.1–184.7), 102.8 (95% UI 61.1–167.3) and 27.8 (95% UI 15.6–41.7) thousand of young people suffered from alcohol use, cannabis use and amphetamine use disorders, respectively, with a rate of 1520 (95% UI 1034–2120), 1180 (95% UI 701–1920) and 320 (95% UI 179 to 478) per 100,000 young people.

In addition, alcohol use, opioid use and amphetamine use disorders were associated with the highest burden in terms of YLDs in Italy in 2019. In particular, 13.6 (95% UI 8.1–21.5), 4.3 (95% UI 2.4–7) and 3.7 (95% UI 1.8–6.4) thousand of YLDs were attributed to alcohol use, opioid use and amphetamine use disorders, respectively, among Italian young people with rates of 155.8 (95% UI 92.5–247.2), 49 (95% UI 27.5–80.9) and 42.2 (95% UI 20.3–73.7) per 100,000 young people.

### Trends of mental health disorders in people aged 10–24 years by sex in Italy

Prevalence and YLDs rates of mental health disorders overall decreased for both sexes between 1990 and 2019 approximately by 2% and 3%, respectively (Table 1).

**Table 1:** Prevalence and years lived with disability (YLDs) for 2019, percentage change of prevalence and YLD counts, and percentage change of age-specific prevalence and YLD rates for 1990–2019 by sex for mental disorders among Italian young people aged 10–24 years (Source: Global Burden of Disease study 2019; generated from data available at http://ghdx.healthdata.org/gbd-results-tool)

	Sex	Prevalence				YLDs			
		2019 age-specific counts (thousands)	Percentage change in age specific counts, 1990–2019	2019 age-specific rate per 100,000 people	Percentage change in age-specific rates, 1990–2019	2019 age-specific counts (thousands)	Percentage change in age-specific counts, 1990–2019	2019 age -specific rate per 100,000 people	Percentage change in age-specific rates, 1990–2019
Mental disorders	M	690.5 (616.9 to 779.9)	− 30.5 (− 33 to − 27.9)	15307.1 (13674.4 to 17288)	− 1.7 (− 5.2 to 2.1)	78.1 (55.6 to 106.3)	− 32 (− 33.7 to − 30.5)	1730.9 (1232.4 to 2355.4)	− 3.8 (− 6.1 to − 1.6)
	F	759.2 (668.7 to 859.5)	− 33.2 (− 34.5 to − 31.8)	18065.1 (15910.3 to 20450.3)	− 2.5 (− 4.4 to − 0.4)	108.8 (75.2 to 149.7)	− 33.3 (− 34.7 to − 31.9)	2588 (1790.4 to 3562.6)	− 2.7 (− 4.7 to − 0.5)
Anxiety disorders	M	217 (170.2 to 276.7)	− 34.5 (− 36.9 to − 31.8)	4810.5 (3772.3 to 6132.7)	− 7.3 (− 10.7 to − 3.6)	21.6 (14.3 to 30.6)	− 34.4 (− 37.4 to − 30.9)	478 (317.4 to 679.2)	− 7.1 (− 11.4 to − 2.2)
	F	397.5 (311.9 to 499.4)	− 33.6 (− 36 to − 31.1)	9458.1 (7422.4 to 11881.9)	− 3 (− 6.5 to 0.6)	39 (26.2 to 55.6)	− 33.5 (− 36.2 to − 30.7)	927.2 (622.7 to 1323.1)	− 2.9 (− 6.8 to 1.2)
Attention-deficit/hyperactivity disorder	M	174.7 (121 to 252.3)	− 24.4 (− 35.1 to − 12.5)	3873 (2682.5 to 5592.7)	7 (− 8.1 to 23.8)	2.1 (1.2 to 3.9)	− 24.2 (− 35.9 to − 12.1)	47.4 (26.2 to 85.7)	7.2 (− 9.3 to 24.3)
	F	35.1 (23.8 to 49.7)	− 31.1 (− 41.9 to − 17.9)	834.8 (566.1 to 1181.6)	0.6 (− 15.2 to 19.8)	0.4 (0.2 to 0.7)	− 31 (− 42.5 to − 17.1)	10.1 (5.5 to 17.5)	0.7 (− 16.1 to 21.1)
Autism spectrum disorders	M	44.9 (37.4 to 53.2)	− 29.1 (− 29.3 to − 29)	996 (829.3 to 1178.3)	0.2 (0 to 0.5)	7 (4.5 to 10)	− 29 (− 31.7 to − 26.3)	154.5 (100.8 to 220.9)	0.4 (− 3.4 to 4.3)
	F	9.4 (7.6 to 11.4)	− 31.3 (− 31.5 to − 31.2)	223.9 (181.7 to 271.6)	0.3 (0 to 0.5)	1.4 (0.9 to 2.1)	− 31.3 (− 36 to − 25.8)	34.3 (22.5 to 50.4)	0.3 (− 6.5 to 8.4)
Bipolar disorders	M	32.2 (24 to 41.7)	− 29.5 (− 31.9 to − 26.5)	713.9 (533 to 923.6)	− 0.3 (− 3.6 to 3.9)	7.2 (4 to 11.3)	− 29.5 (− 34.2 to − 24.5)	160.4 (89.7 to 249.7)	− 0.3 (− 6.9 to 6.8)
	F	41.4 (31.1 to 53.2)	− 32 (− 34.1 to − 29.4)	983.9 (739 to 1265.6)	− 0.7 (− 3.8 to 3.1)	9.1 (5.2 to 14.2)	− 31.9 (− 35.6 to − 27.8)	217.2 (123.4 to 337.6)	− 0.6 (− 5.9 to 5.4)
Conduct disorder	M	112.4 (83.8 to 149.5)	− 25.3 (− 26.5 to − 24.3)	2491.4 (1858.6 to 3313.7)	5.7 (4 to 7.1)	13.7 (7.8 to 21.7)	− 25.3 (− 27.6 to − 22.7)	303.4 (173.9 to 480.2)	5.7 (2.4 to 9.3)
	F	66.1 (44.9 to 91.6)	− 26.1 (− 27.8 to − 24.8)	1572.1 (1069.2 to 2180.4)	7.9 (5.5 to 9.8)	8 (4.3 to 12.9)	− 26 (− 29.4 to − 22.6)	189.4 (103.3 to 306.3)	8.1 (3 to 13)
Depressive disorders	M	95.5 (74.4 to 120.4)	− 35.9 (− 38.7 to − 32.8)	2117.3 (1648.6 to 2668.6)	− 9.3 (− 13.2 to − 4.9)	17.4 (11.1 to 25.9)	− 36.4 (− 39.2 to − 33.1)	385.6 (246.8 to 575.1)	− 10.1 (− 14 to − 5.4)
	F	174 (135.4 to 219.4)	− 33.1 (− 35.5 to − 30.6)	4140.9 (3220.9 to 5219.4)	− 2.4 (− 5.8 to 1.4)	32.7 (20.7 to 48.6)	− 32.8 (− 35.4 to − 30.2)	776.9 (491.5 to 1156.6)	− 2 (− 5.7 to 1.9)
Eating Disorders	M	13.5 (9.1 to 18.9)	− 27 (− 32.7 to − 20.7)	299 (201.3 to 419)	3.2 (− 4.7 to 12.2)	2.9 (1.7 to 4.6)	− 26.9 (− 32.5 to − 20.6)	64.6 (38 to 102)	3.4 (− 4.5 to 12.4)
	F	63.7 (44.7 to 90.1)	− 37.4 (− 41 to − 33.6)	1514.6 (1063.4 to 2144.6)	− 8.5 (− 13.8 to − 3)	13.6 (7.7 to 21.3)	− 37.4 (− 41 to − 33.5)	323.4 (184.1 to 506.5)	− 8.5 (− 13.9 to − 3)
Idiopathic developmental intellectual disability	M	19.4 (3.9 to 35)	− 38 (− 47.2 to − 35.5)	429.9 (87.5 to 775.6)	− 12.3 (− 25.2 to − 8.7)	0.9 (0.2 to 1.8)	− 37.6 (− 45.7 to − 32.5)	20.7 (5.4 to 39)	− 11.7 (− 23.2 to − 4.5)
	F	21.9 (9.6 to 34.2)	− 41.9 (− 48.3 to − 37.9)	520.8 (227.4 to 812.9)	− 15.1 (− 24.5 to − 9.4)	1.1 (0.5 to 1.8)	− 40.1 (− 45 to − 35.4)	25 (11.1 to 41.7)	− 12.5 (− 19.7 to − 5.6)
Schizophrenia	M	4.4 (3.1 to 5.9)	− 35.2 (− 37.8 to − 32.7)	96.7 (69.4 to 131.1)	− 8.3 (− 12 to − 4.8)	2.9 (1.9 to 4.4)	− 34.9 (− 42.5 to − 25.9)	65.1 (42.3 to 96.5)	− 7.9 (− 18.6 to 4.8)
	F	3.5 (2.5 to 4.7)	− 37.5 (− 39.7 to − 35.3)	83.5 (60 to 112.7)	− 8.7 (− 11.9 to − 5.5)	2.3 (1.5 to 3.4)	− 37.5 (− 44.6 to − 29)	54.8 (35.8 to 81.1)	− 8.7 (− 19.2 to 3.7)
Other mental disorders	M	29.8 (18.3 to 43.5)	− 33.2 (− 33.3 to − 33.2)	661.4 (405.4 to 964)	− 5.5 (− 5.6 to − 5.5)	2.3 (1.2 to 3.8)	− 33.2 (− 38.6 to − 27.4)	51.2 (26.4 to 84.1)	− 5.5 (− 13.2 to 2.7)
	F	16.3 (9.2 to 25)	− 36.1 (− 36.2 to − 36.1)	388.8 (220 to 594.5)	− 6.7 (− 6.8 to − 6.7)	1.2 (0.6 to 2.1)	− 36 (− 43 to − 28.3)	29.7 (15.1 to 49.8)	− 6.6 (− 16.8 to 4.7)

**Fig. 1:**
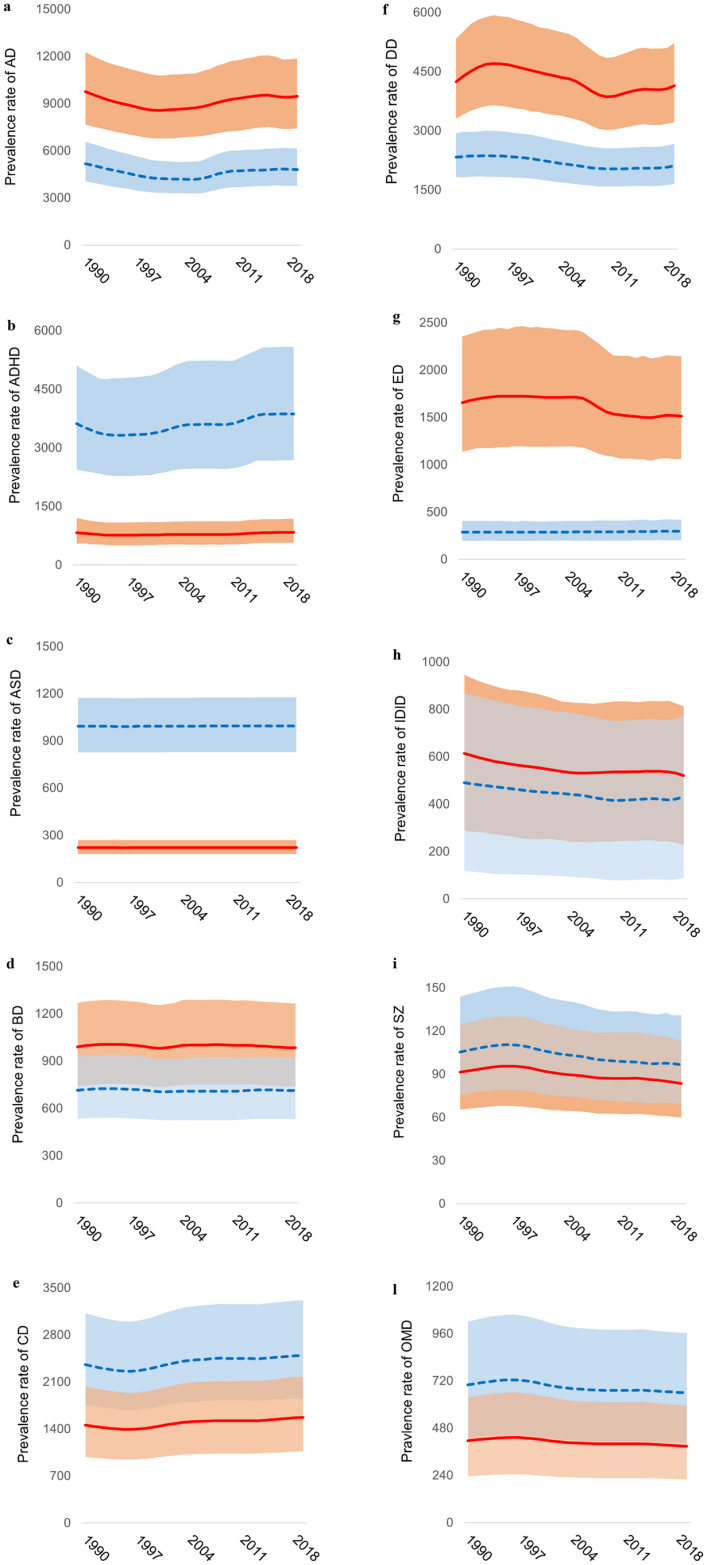
Trends in prevalence rates (per 100,000 young people aged 10–24 years) of **a** anxiety disorders, (AD) **b** attention-deficit/hyperactivity disorder (ADHD), **c** autism spectrum disorders (ASD), **d** bipolar disorders (BD), **e** conduct disorder (CD), **f** depressive disorders (DD), **g** eating disorders (ED), **h** idiopathic developmental intellectual disability (IDID), **i** schizophrenia (SZ) and **l** other mental disorders (OMD) by sex (Male: blue dotted line; Female: solid red line)

Figure 1 and Online Resource 3 show trends in prevalence and YLDs rates, respectively, between 1990 and 2019. Prevalence and YLDs rates of anxiety and depressive disorders, idiopathic developmental intellectual disability, schizophrenia and other mental disorders consistently decreased over time (Table 1). The largest negative percentual changes between 1990 and 2019 were observed for idiopathic developmental intellectual disability. Eating disorders rates decreased in females while increased in males. In addition, prevalence and YLDs rates of conduct and attention-deficit/hyperactivity disorders consistently increased over time (except for rates of attention-deficit/hyperactivity disorder among females). Prevalence rates of conduct disorder increased by 5.7% (95% UI 4–7.1) in males and 7.9% (95% UI 5.5–9.8) in females between 1990 and 2019, while the diagnosis of attention-deficit/hyperactivity disorder increased by 7% (95% UI -8.1–23.8) (with similar changes for YLDs rates). Finally, rates of autism spectrum and bipolar disorders were mainly stable over time.

### Trends of substance use disorders in people aged 10–24 years by sex in Italy

Prevalence and YLDs rates of substance use disorders overall largely decreased for both sexes between 1990 and 2019 approximately by 25% (Table 2).

**Table 2:** Prevalence and years lived with disability (YLDs) for 2019, percentage change of prevalence and YLD counts, and percentage change of age-specific prevalence and YLD rates for 1990–2019 by sex for substance use disorders among Italian young people aged 10–24 years (Source: Global Burden of Disease study 2019; generated from data available at http://ghdx.healthdata.org/gbd-results-tool)

	Sex	Prevalence				YLDs			
		2019 age-specific counts (thousands)	Percentage change in age specific counts, 1990–2019	2019 age-specific rate per 100,000 people	Percentage change in age-specific rates, 1990–2019	2019 age-specific counts (thousands)	Percentage change in age-specific counts, 1990–2019	2019 age-specific rate per 100,000 people	Percentage change in age-specific rates, 1990–2019
Substance use disorders	M	181.7 (141.2 to 232.4)	− 47.1 (− 51.6 to − 42.6)	4028.1 (3131.1 to 5151.7)	− 25.1 (− 31.5 to − 18.8)	19.3 (12.8 to 27.3)	− 49.2 (− 53.9 to − 45.1)	427.5 (284.1 to –604.6)	− 28.1 (− 34.7 to − 22.3)
	F	96.7 (75.4 to 124.5)	− 48.6 (− 52.9 to − 44.3)	2301.6 (1794.5 to 2961.6)	− 24.9 (− 31.2 to − 18.7)	10.5 (6.9 to 14.5)	− 43.3 (− 47.2 to − 38.9)	249.2 (165. to –344.7)	− 17.2 (− 22.8 to − 10.8)
Alcohol use disorders	M	90.6 (61.6 to 127.3)	− 32.6 (− 35.3 to − 29.6)	2007.3 (1366.1 to 2823)	− 4.7 (− 8.5 to − 0.4)	9.3 (5.5 to 14.7)	− 32.5 (− 36.7 to − 28.1)	206.8 (122.7 to 326)	− 4.6 (− 10.4 to 1.8)
	F	41.9 (28.4 to 57.9)	− 34.6 (− 37.1 to − 32.2)	997.7 (675.5 to 1377.3)	− 4.5 (− 8.2 to − 1)	4.3 (2.5 to 6.7)	− 34.5 (− 38.5 to − 29.8)	101.1 (60.3 to 159.7)	− 4.4 (− 10.2 to 2.5)
Drug use disorders	M	95.3 (65.4 to 137.7)	− 56.1 (− 61.5 to − 50.1)	2112.9 (1448.9 to 3052.3)	− 37.9 (− 45.6 to − 29.4)	10 (6.4 to 14.5)	− 58.7 (− 63.7 to − 53.7)	220.7 (141.8 to 320.8)	− 41.6 (− 48.6 to − 34.5)
	F	56 (40 to 80.4)	− 55.7 (− 60.1 to − 50.3)	1333.3 (952.9 to 1912.6)	− 35.3 (− 41.7 to − 27.5)	6.2 (3.9 to 9)	− 48.1 (− 53.4 to − 41.8)	148.1 (92.7 to 214.2)	− 24.2 (− 32 to − 15)
Amphetamine use disorders	M	17.5 (9.7 to 26.3)	− 45.1 (− 48.3 to − 41.3)	388 (215.8 to 583.2)	− 22.3 (− 26.8 to − 16.9)	2.3 (1.1 to 4.1)	− 45 (− 51 to − 38)	51.7 (24.9 to 90.2)	− 22.2 (− 30.7 to − 12.3)
	F	10.3 (5.7 to 15.6)	− 48.1 (− 51.2 to − 44.1)	246.1 (135.4 to 370.3)	− 24.2 (− 28.8 to − 18.3)	1.3 (0.6 to 2.3)	− 47.9 (− 55 to − 39.9)	32.1 (15.1 to 55)	− 24 (− 34.4 to − 12.3)
Cannabis use disorders	M	65.5 (37.3 to 107.9)	− 57.7 (− 65.1 to − 49.6)	1451.1 (826.8 to 2391.4)	− 40.2 (− 50.6 to − 28.8)	1.9 (0.9 to 3.6)	− 57.7 (− 65.7 to − 49.3)	42.5 (19.8 to 80.9)	− 40.1 (− 51.5 to − 28.3)
	F	37.4 (22.5 to 60.7)	− 59.8 (− 65.5 to − 53.5)	889.1 (536 to 1444.3)	− 41.3 (− 49.6 to − 32.1)	1.1 (0.5 to 2)	− 59.8 (− 66.6 to − 52.1)	25.7 (12.6 to 47.4)	− 41.3 (− 51.2 to − 30)
Cocaine use disorders	M	8 (4.9 to 12.1)	− 27.8 (− 32.3 to − 22.7)	176.5 (108.8 to 268.1)	2.2 (− 4.2 to 9.4)	1.1 (0.6 to 1.9)	− 27.8 (− 38.9 to − 15.1)	24.6 (12.3 to 42)	2.2 (− 13.6 to 20)
	F	4.2 (2.5 to 6.5)	− 27.5 (− 32.4 to − 22)	99.5 (58.5 to 154.7)	5.8 (− 1.3 to 13.9)	0.6 (0.3 to 1)	− 27.4 (− 41.2 to − 10.7)	13.6 (6.5 to 24.1)	6.1 (− 14.2 to 30.3)
Opioid use disorders	M	5.4 (3.5 to 8)	− 76.9 (− 81.4 to − 72.2)	119.6 (78 to 177.1)	− 67.2 (− 73.7 to − 60.7)	2.3 (1.3 to 3.8)	− 76.8 (− 81.9 to − 71.7)	51.5 (28.9 to 83.6)	− 67.2 (− 74.4 to − 60)
	F	4.6 (2.9 to 7.4)	− 47.3 (− 58 to − 35.1)	110.1 (68.1 to 175.4)	− 23.1 (− 38.7 to − 5.2)	1.9 (1.1 to 3.4)	− 47.1 (− 59.6 to − 34.2)	46.4 (25 to 80)	− 22.8 (− 41.1 to − 4)
Other drug use disorders	M	0.4 (0.2 to 0.6)	− 28.1 (− 34.2 to − 21.6)	8.4 (5.3 to 12.7)	1.7 (− 6.8 to 10.9)	2.3 (1.3 to 3.7)	− 40.3 (− 48.3 to − 30.4)	50.4 (28.3 to 81.9)	− 15.5 (− 26.9 to − 1.5)
	F	0.2 (0.1–0.4)	− 35.3 (− 39.7 to − 30.9)	5.7 (3.3 to 9.1)	− 5.5 (− 12 to 0.9)	1.3 (0.7 to 2)	− 42.9 (− 51.2 to − 32.3)	30.3 (16.6 to 48.2)	− 16.7 (− 28.7 to − 1.2)

**Fig. 2:**
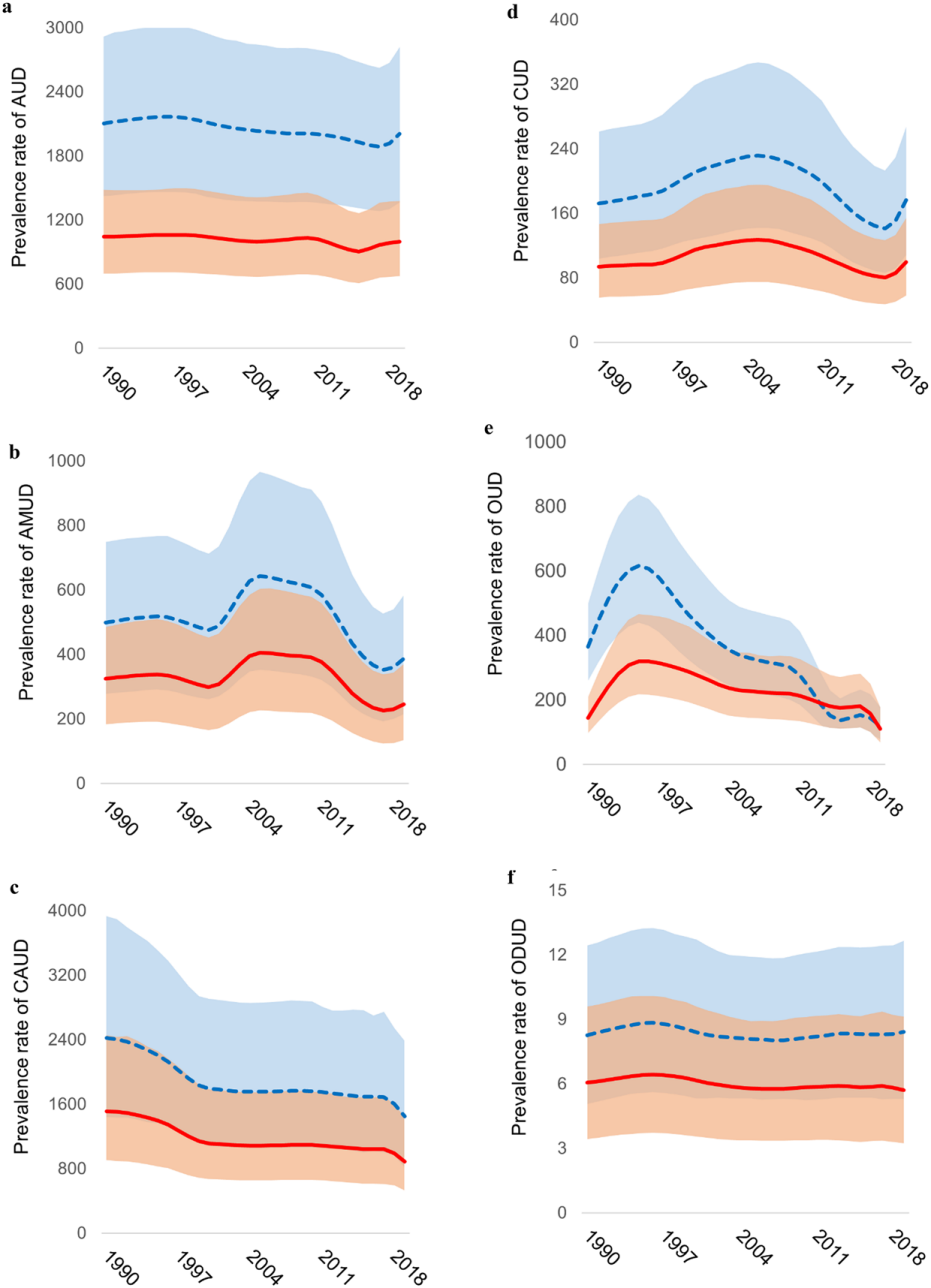
Trends in prevalence rates (per 100,000 young people aged 10–24 years) of **a** alcohol use disorders (AUD), **b** amphetamine use disorder (AMUD), **c** cannabis use disorders (CAUD), **d** cocaine use disorders (CUD), **e** opioid use disorders (OUD) and **f** other drug use disorders (ODUD) by sex (Male: blue dotted line; Female: solid red line)

Figure 2 and Online Resource 4 show trends in prevalence and YLDs rates of specific substance use disorders, respectively, between 1990 and 2019. Prevalence and YLDs rates of alcohol use, amphetamine use, cannabis use, opioid use and other drug use disorders consistently decreased between the two time points (except for other drug use in males) (Table 2). The largest negative percentual changes were observed for cannabis use, opioid use and amphetamine use disorders. Prevalence rates of cocaine use disorders increased by 2.2% (95% UI − 4.2–9.4) in males and 5.8% (95% UI − 1.3–13.9) in females between 1990 and 2019 (with similar changes for YLDs rates).

### Mental health disorders in 2019 according to age group and sex in Italy

**Fig. 3:**
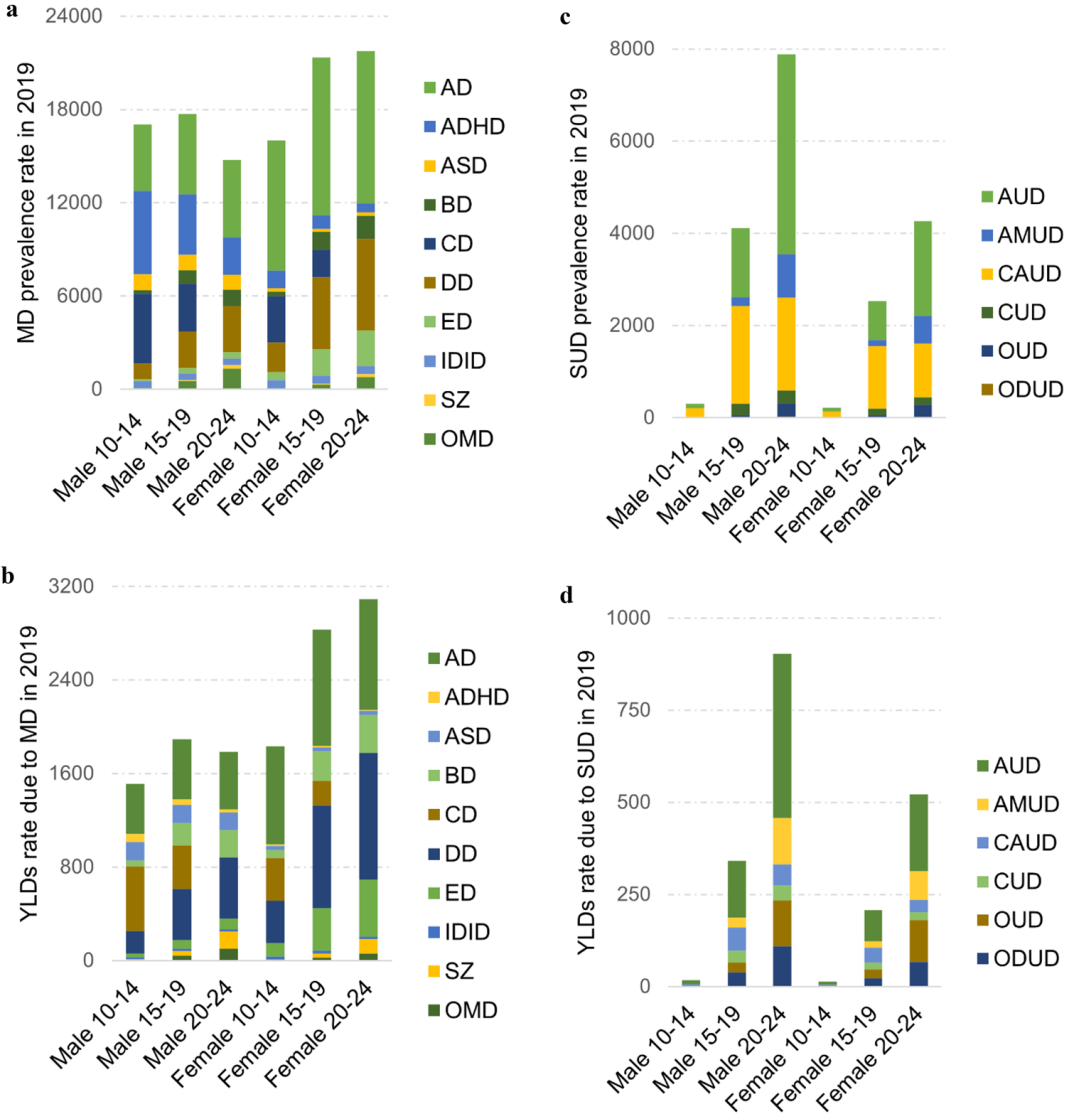
**a** Prevalence rate and **b** YLDs rate of mental disorders (MD) as well as **c** prevalence rate and **d** YLDs rate of substance use disorders (SUD) by age group and sex. *AD* anxiety disorders, *ADHD* attention-deficit/hyperactivity disorder, *ASD* autism spectrum disorders, *BD* bipolar disorders, *CD* conduct disorder, *DD* depressive disorders, *ED* eating disorders, *IDID* idiopathic developmental intellectual disability, *SZ* schizophrenia, *OMD* other mental disorders, *AUD* alcohol use disorders, *AMUD* amphetamine use disorders, *CAUD* cannabis use disorders, *CUD* cocaine use disorders, *OUD* opioid use disorders, *ODUD* other drug use disorders

Mental health disorders were more commonly diagnosed among females aged 15–19 and 20–24 years than males of the same age groups (Fig. 3a; Online Resource 5). Specifically, prevalence rates of mental health disorders were nearly the same across sexes during early adolescence despite females suffered from higher YLDs compared to males (Fig. 3b; Online Resource 5). The difference in prevalence and YLDs rates of mental health disorders between sexes increased with age with females being at higher risk than males. Prevalence rates (per 100,000 young people) of mental health disorders were 13.8 (95% UI 12.1–15.9) thousand among males and 19.8 (95% UI 17.1–22.8) thousand among females, while YLDs rates were 1.8 (95% UI 1.2–2.4) thousand for males and 3.1 (95% UI 2.2–4.2) thousand for females. Anxiety and depressive disorders were related to the highest levels of disability in terms of YLDs across both sexes, while conduct and eating disorders were associated with high rates of YLDs for males and females, respectively.

### Substance use disorders in 2019 according to age group and sex in Italy

Prevalence rates of substance use disorders were very low during early adolescence (Male: 280.8 (95% UI 138–490.9); Female: 194.1 (95% UI 113.2–308.2)) while increased with age (Fig. 3c; Online Resource 6). Males were more likely diagnosed with substance use disorders than females. In particular, prevalence rate of substance use disorders was nearly twice as high in males (7.6 thousand per 100,000) than females (4.2 thousand) aged 20–24 years. Moreover, males showed higher YLDs due to substance use disorders as compared to females (Fig. 3d). Alcohol use disorders were associated to the highest levels of disability in terms of YLDs. Amphetamine use and opioid use disorders were also related to high levels of burden especially in young adulthood.

## Discussion

The present study provides detailed information on the burden of mental health and substance use disorders in terms of prevalence and YLDs rates as well as on changes occurred between 1990 and 2019 among Italian young people aged 10–24 years. This is the first study to describe the burden estimates of mental health and substance use disorders in Italian young people based on the GBD 2019 data. In 2019, there were 1.4 (95% UI 1.3–1.6) and 0.3 (95% UI 0.2–0.4) millions of young people suffering from mental health and substance use disorders, respectively, in Italy, with a rate of 16637 (95% UI for mental health disorders: 14880–18701) and 3195 (95% UI for substance use disorders: 2487–4072) per 100,000 young people. Overall, percentage changes between 1990 and 2019 showed decreases in diseases burden, especially for substance use disorders. These findings are in line with those of a recent study indicating that mental health of young people improved substantially both globally and in Europe [8]. In addition, a possible explanation for the results presented could be related to the high quality of the Italian health system that ensures universal coverage for their citizens [14] despite previous studies suggested that the use of health services was relatively scarce or unsatisfactory among Italian adults of the general population with mental health disorders [21–24], while there is a lack of data on Italian young people. However, mental health and substance use disorders are still among the most significant leading cause of disease burden among young people, in particular, in high-income countries [6, 9, 10]. Attention-deficit/hyperactivity disorder for males, eating disorders for females, and anxiety, depressive and conduct disorders for both sexes, affected between 1.5 (eating disorders among female sex) and 9.5 thousand (anxiety disorders among female sex) Italian youths per 100,000 young people in 2019. The highest disease burdens in terms of YLDs were due to anxiety, bipolar, depressive and conduct disorders for both sexes and to eating disorders for females (between 160 YLDs due to bipolar disorders among males and 927 YLDs due to anxiety among females per 100,000 young people). Regarding substance use disorders, their prevalence and YLDs rates were generally lower than those of mental health disorders. Alcohol and drug use disorders impacted on the lives of one-two thousand (per 100,000 young people) Italian youths, and they caused between 101 and 220 YLDs (per 100,000 young people), with higher rates among males than among females. Importantly, between 1990 and 2019, diagnoses of attention-deficit/hyperactivity, autism spectrum, conduct and eating (among males) disorders increased as well as cocaine use disorder. These results are in line with those observed in western Europe and globally with few exception [7, 25, 26]. Diagnostic substitution and confounders may exert some role in the increase of prevalence rates of attention-deficit/hyperactivity and autism spectrum disorders [27, 28]. It is also difficult to clarify whether the increase in prevalence rates reflect a true change or an increased discovery rate [27]. Finally, it should be noted that despite the burden of depressive disorders decreased between 1990 and 2019, the rates have increased after 2010. Similarly, positive trends have been observed in rates of alcohol and amphetamine use disorders during the years just before 2019.

Mental health disorders were more commonly diagnosed among females than males with the differences between sexes becoming evident during middle-late adolescence. Prevalence rate of mental health disorders increased with age among females while it was mainly stable among males. Anxiety and depressive disorders were related to the highest levels of disability in terms of YLDs across both sexes, while conduct disorders and eating disorders were associated with high rates of YLDs for males and females, respectively. These results are in line with those of the previous studies among Italian adolescents and adults pointing out that depression and anxiety were the most common mental health disorders and females at higher risk than males [21, 29, 30]. Frigerio et al. [30] conducted an epidemiological study to evaluate prevalence and correlates of mental health disorders among 3418 Italian adolescents. The authors showed that approximately 10% of adolescents was affected by symptoms of psychopathology, especially emotional disorders, and that the prevalence estimates among females increased significantly with age [30]. Moreover, these findings have been confirmed by other studies conducted in European as well as non-European countries [31–34].

Regarding substance use disorders, males showed higher rates than females during adolescence and young adulthood. Alcohol use disorders were associated to the highest levels of disability in terms of YLDs, while amphetamine use and opioid use disorders were related to high levels of burden especially in young adulthood. Italian GBD 2019 data are in line with the findings of previous studies conducted in Italy indicating male sex as a risk factor of substance use and that it increases with age [35–38]. Molinaro et al. [38] examined trends in illegal substance use of Italian adolescents between 1999 and 2009. The authors reported decreases in cannabis, cocaine and heroin use, while increases in hallucinogens and stimulants over time. GBD 2019 data indicated a consistent decrease in substance use disorders in Italian young people between 1990 and 2019 except for cocaine use disorders. It has been proposed that the decline in prevalence of adolescent substance use disorders and delinquent behaviours may reflect a decreasing trend in an underlying externalizing-like trait of psychopathology related to disinhibition or risk preferences [39]. In the present study, cocaine use disorders increased by 2.2% and 5.8% in male and female gender, respectively, with these changes probably due to the sudden increase in rates during the years just before 2019 (the same applies to positive trends in rates of alcohol and amphetamine use disorders). Despite caution is needed in interpreting these findings on cocaine use disorders considering the wide confidence intervals of the percentual changes in rates between 1990 and 2019 in Italy, the rate of cocaine use disorder consistently increased in western Europe over the same time period (Online Resource 2). The upward trend may be related to the increased cocaine availability in Europe that was at an all-time high in 2017 [40]. Cocaine prevalence and treatment demands have significantly increased in European adults since 2015, probably due to increase in cocaine production, its wide availability and the development of new purchasing methods [41]. Those findings are of particular concern in view of an high mortality risk for cocaine users under 25 years of age [42]. Finally, cannabis legalization has been the object of a controversial debate in Italy [43] and the long-term public health impacts of a possible legalization should be monitored [44].

The results of the present study point out an important challenge for the national health system, required to take care of the psychophysical health of young people. Some authors noted that while identifying and treating mental health and substance use disorders is desirable, effective preventive strategies are more likely to reduce the burden associated with these disorders [7]. Thus, the national health system should continuously sustain efforts in mental health promotion and prevention. Every public health system that can truly be described as functioning must focus its attention and investment on the mental health of young people. At the same time, increased treatment rates do not seem to be accompanied by decreases in prevalence rates of common mental disorders [45, 46]. Therefore, community-level preventive interventions addressing social and economic determinants are needed to promote and sustain mental health [46–48]. Recently, Sinha et al. [49] have discussed the citiesRISE experience, a network approach to young people’s mental health based on collective action. The citiesRISE approach constitutes a broad initiative that aims to mobilize all available resources to meet mental health needs of populations through the involvement of multiple stakeholders and organization at the local, national and global levels, as well as interventions addressing supply and demand for services and relevant social factors [49].

Some limitations of the study should be considered in interpreting the results. First, the estimates presented rely on secondary data and, then, on the quality and quantity of primary data sources [7, 50]. However, the majority of high-income regions had some level of data coverage for mental health disorders in children and adolescents, with a coverage of the available prevalence data of 16.3% in Western Europe [51]. Second, changes in coding practices or coding systems may influence the estimates, despite GBD 2019 applies adjustments to the data accounting for different coding systems or case definitions. Third, disability weights may present possible sources of error that impact YLDs estimates [8]. Previous studies discussed widely and in detail GBD study limitations [6, 10, 18]. Despite the above limitations, GBD 2019 data provide useful syntheses of the available evidence on mental health of young people.

## Conclusion

Age-specific prevalence and YLDs rates of mental health and substance use disorders showed negative trends (decreasing patterns) overall between 1990 and 2019 among young people in Italy. However, some exceptions exist. Diagnoses of attention-deficit/hyperactivity, autism spectrum, conduct and eating (among males) disorders increased as well as cocaine use disorder. Moreover, trends in rates of depressive disorders have increased after 2010 as well as trends in rates of alcohol and amphetamine use disorders during the years just before 2019. Mental health and substance use disorders are still among the most significant leading cause of disease burden. The highest levels of disability in terms of YLDs were due to anxiety, depressive, conduct and eating disorders, and alcohol use, amphetamine use and opioid use disorders. The burden of diseases was higher among females than males for mental health disorders, among males as compared to females for substance use disorders, and in middle-late adolescence and young adulthood than early adolescence. Preventive interventions at the community level are needed to promote and sustain mental health of young people.

## Supplementary Information

Below is the link to the electronic supplementary material.

Supplementary file1 (DOCX 24 KB)

Supplementary file2 (DOCX 21 KB)

Supplementary file3 (DOCX 880 KB)

Supplementary file4 (DOCX 601 KB)

Supplementary file5 (DOCX 20 KB)

Supplementary file6 (DOCX 18 KB)

## Data Availability

Data visualisation tools, from which the data were extracted, are publicly available online (http://ghdx.healthdata.org/gbd-results-tool).
