# Supplementary material for: Burden of mental health and substance use disorders among Italian young people aged 10–24 years: results from the Global Burden of Disease 2019 Study
Source: Soc Psychiatry Psychiatr Epidemiol. 2022 Jan 20;57(4):683–94. doi: 10.1007/s00127-022-02222-0 (PMC8960651; doi:10.1007/s00127-022-02222-0)
Supplement: Supplementary file 1 — Supplementary file1 (DOCX 24 KB) [file 127_2022_2222_MOESM1_ESM.docx]

**Online Resource 1**

Prevalence and years lived with disability (YLDs) for 2019, percentage change of prevalence and YLD counts, and percentage change of age-specific prevalence and YLD rates for 1990-2019 for both sexes combined for mental disorders among young people aged 10-24 years in Italy, Western Europe and globally (Source: Global Burden of Disease study 2019; generated from data available at <http://ghdx.healthdata.org/gbd-results-tool>)

|  | **Prevalence** |  |  |  |  | **YLDs** |  |  |  |
| --- | --- | --- | --- | --- | --- | --- | --- | --- | --- |
|  | **2019 age-specific counts (thousands)** | **Percentage change in age specific counts, 1990-2019** | **2019 age -specific rate per 100,000 people** | **Percentage change**  **in age-specific**  **rates, 1990-2019** |  | **2019 age-specific counts (thousands)** | **Percentage change in age-specific counts, 1990-2019** | **2019 age -specific rate per 100,000 people** | **Percentage change**  **in age-specific**  **rates, 1990-2019** |
| **Globally** |  |  |  |  |  |  |  |  |  |
| *Mental disorders* | 248030.9 (221174.5 to 279453.5) | 17.6 (16.4 to 18.8) | 13321.6 (11879.1 to 15009.3) | -2.2 (-3.2 to -1.1) |  | 28163.6 (20082.6 to 38084.1) | 20.7 (19.4 to 22) | 1512.7 (1078.6 to 2045.5) | 0.4 (-0.7 to 1.5) |
| Anxiety disorders | 75874.4 (57045.1 to 99769) | 17.9 (15.7 to 20) | 4075.2 (3063.9 to 5358.5) | -1.9 (-3.8 to -0.2) |  | 7497.9 (4950.3 to 10835.4) | 17.9 (15.7 to 20.3) | 402.7 (265.9 to 582) | -1.9 (-3.8 to 0.1) |
| Attention-deficit/hyperactivity disorder | 42107.3 (29737.6 to 57646.2) | 6.7 (4 to 9.4) | 2261.6 (1597.2 to 3096.1) | -11.3 (-13.5 to -9) |  | 514.4 (288.3 to 898.1) | 6.7 (3.8 to 9.8) | 27.6 (15.5 to 48.2) | -11.2 (-13.6 to -8.6) |
| Autism spectrum disorders | 7389.3 (6121.4 to 8824) | 19.6 (19.1 to 20.1) | 396.9 (328.8 to 473.9) | -0.5 (-0.9 to -0.1) |  | 1145.2 (746.7 to 1670.1) | 19.7 (18.3 to 21.1) | 61.5 (40.1 to 89.7) | -0.4 (-1.6 to 0.7) |
| Bipolar disorders | 8962.4 (6443 to 12010.6) | 27.6 (25.5 to 29.8) | 481.4 (346 to 645.1) | 6.2 (4.4 to 8) |  | 2004.9 (1085.3 to 3224.7) | 27.9 (25.3 to 30.3) | 107.7 (58.3 to 173.2) | 6.4 (4.3 to 8.4) |
| Conduct disorder | 33407.8 (23686.6 to 45284.1) | 24.5 (22.3 to 26.7) | 1794.3 (1272.2 to 2432.2) | 3.6 (1.7 to 5.4) |  | 4055 (2241 to 6476.9) | 24.7 (22.2 to 27) | 217.8 (120.4 to 347.9) | 3.8 (1.7 to 5.7) |
| Depressive disorders | 46000.7 (36577.5 to 57783.7) | 21.7 (18.7 to 24) | 2470.7 (1964.6 to 3103.5) | 1.2 (-1.3 to 3.2) |  | 8426.5 (5457.7 to 12502.7) | 20.7 (17.4 to 23.5) | 452.6 (293.1 to 671.5) | 0.4 (-2.4 to 2.7) |
| Eating Disorders | 5160.8 (3642.4 to 7020.8) | 40.3 (37.8 to 43) | 277.2 (195.6 to 377.1) | 16.7 (14.7 to 18.9) |  | 1108.4 (654.3 to 1723.8) | 40.5 (38 to 43.1) | 59.5 (35.1 to 92.6) | 16.9 (14.8 to 19.1) |
| Idiopathic developmental intellectual disability | 35349.2 (22324.7 to 48753.5) | 12.9 (10.3 to 14.6) | 1898.6 (1199 to 2618.5) | -6 (-8.2 to -4.7) |  | 1424.6 (747 to 2339.7) | 12.4 (8 to 15.8) | 76.5 (40.1 to 125.7) | -6.5 (-10.2 to -3.7) |
| Schizophrenia | 1927.8 (1392.5 to 2627.6) | 15.6 (11.9 to 18.3) | 103.5 (74.8 to 141.1) | -3.9 (-6.9 to -1.5) |  | 1293.1 (844.7 to 1922.1) | 15.8 (11.6 to 19.3) | 69.5 (45.4 to 103.2) | -3.7 (-7.2 to -0.8) |
| Other mental disorders | 8970 (5641.3 to 12972.9) | 21.6 (20.7 to 22.5) | 481.8 (303 to 696.8) | 1.1 (0.4 to 1.9) |  | 693.7 (362.6 to 1139.4) | 21.7 (19.3 to 24.1) | 37.3 (19.5 to 61.2) | 1.3 (-0.7 to 3.2) |
|  |  |  |  |  |  |  |  |  |  |
| **Western Europe** |  |  |  |  |  |  |  |  |  |
| *Mental disorders* | 12407.7 (10952.9 to 14146.9) | -13.1 (-14.9 to -11.2) | 17333.7 (15301.3 to 19763.4) | -0.3 (-2.3 to 1.9) |  | 1584.5 (1109.8 to 2161.2) | -13.8 (-15.9 to -11.7) | 2213.5 (1550.4 to 3019.2) | -1.1 (-3.4 to 1.4) |
| Anxiety disorders | 5137.9 (3800.3 to 6852.4) | -11.9 (-15.3 to -8) | 7177.7 (5309 to 9572.9) | 1.2 (-2.7 to 5.6) |  | 506 (329.1 to 739.4) | -11.8 (-15.4 to -7.7) | 706.9 (459.8 to 1033) | 1.3 (-2.9 to 5.9) |
| Attention-deficit/hyperactivity disorder | 1983.9 (1399.1 to 2743.4) | -7.3 (-12.3 to -1.9) | 2771.5 (1954.6 to 3832.6) | 6.4 (0.7 to 12.7) |  | 24.2 (13.3 to 42.1) | -7.3 (-12.9 to -1.2) | 33.8 (18.6 to 58.8) | 6.5 (0 to 13.5) |
| Autism spectrum disorders | 454.2 (382.6 to 536.1) | -11.8 (-12.9 to -10.7) | 634.5 (534.4 to 748.9) | 1.3 (0 to 2.6) |  | 70.2 (46.2 to 100.3) | -11.8 (-14.4 to -9.3) | 98.1 (64.6 to 140.2) | 1.3 (-1.8 to 4.1) |
| Bipolar disorders | 663.2 (480.7 to 882.8) | -14.1 (-16.3 to -11.6) | 926.6 (671.6 to 1233.3) | -1.4 (-3.9 to 1.5) |  | 147.5 (80.2 to 237.6) | -14.1 (-17.5 to -10.6) | 206.1 (112.1 to 332) | -1.4 (-5.3 to 2.7) |
| Conduct disorder | 1386.9 (989 to 1861.4) | -8.1 (-9.2 to -6.8) | 1937.6 (1381.6 to 2600.4) | 5.5 (4.2 to 7) |  | 168.1 (94.1 to 269) | -8 (-10.4 to -5.6) | 234.9 (131.5 to 375.8) | 5.6 (2.9 to 8.4) |
| Depressive disorders | 2425.1 (1916.6 to 3070.2) | -18.3 (-23.5 to -12.8) | 3388 (2677.5 to 4289.1) | -6.2 (-12.2 to 0.1) |  | 452.5 (289.9 to 673.6) | -18.5 (-24.5 to -12.4) | 632.1 (405.1 to 941.1) | -6.4 (-13.4 to 0.5) |
| Eating Disorders | 564.4 (404.8 to 774.1) | -5.2 (-10.3 to -0.6) | 788.5 (565.6 to 1081.4) | 8.8 (3 to 14.1) |  | 120.8 (72 to 186.3) | -5.2 (-10.3 to -0.6) | 168.7 (100.6 to 260.2) | 8.9 (3 to 14.2) |
| Idiopathic developmental intellectual disability | 369.7 (140.3 to 609.1) | -37 (-47.5 to -32.9) | 516.4 (196 to 850.8) | -27.7 (-39.7 to -23) |  | 17.3 (6.7 to 30.6) | -36.9 (-46.2 to -32.5) | 24.2 (9.3 to 42.8) | -27.6 (-38.3 to -22.5) |
| Schizophrenia | 62.8 (44 to 88.6) | -19.8 (-22.8 to -17.3) | 87.8 (61.4 to 123.7) | -7.9 (-11.4 to -5.1) |  | 41.8 (26.7 to 64.3) | -19.5 (-26.5 to -12.8) | 58.4 (37.4 to 89.8) | -7.6 (-15.7 to 0.1) |
| Other mental disorders | 468 (297.1 to 661.2) | -16.7 (-16.8 to -16.6) | 653.8 (415.1 to 923.7) | -4.4 (-4.5 to -4.2) |  | 36 (19 to 58.1) | -16.7 (-21.7 to -11.6) | 50.3 (26.6 to 81.1) | -4.4 (-10.1 to 1.5) |
|  |  |  |  |  |  |  |  |  |  |
| **Italy** |  |  |  |  |  |  |  |  |  |
| *Mental disorders* | 1449.7 (1296.6 to 1629.5) | -32 (-33.2 to -30.6) | 16637.3 (14880.2 to 18700.8) | -2.3 (-4.1 to -0.2) |  | 186.9 (131.2 to 255.9) | -32.8 (-33.9 to -31.6) | 2144.3 (1505.9 to 2937.2) | -3.5 (-5.1 to -1.8) |
| Anxiety disorders | 614.5 (482.5 to 773.9) | -33.9 (-35.8 to -31.9) | 7052 (5537.6 to 8880.9) | -5 (-7.7 to -2.1) |  | 60.5 (40.7 to 86.4) | -33.8 (-36 to -31.6) | 694.6 (466.7 to 992.1) | -4.9 (-8.1 to -1.7) |
| Attention-deficit/hyperactivity disorder | 209.8 (146 to 297.5) | -25.6 (-34.9 to -15.6) | 2407.7 (1675.7 to 3414.1) | 6.9 (-6.4 to 21.3) |  | 2.6 (1.4 to 4.6) | -25.5 (-34.9 to -15.4) | 29.4 (16.1 to 52.7) | 7.1 (-6.5 to 21.5) |
| Autism spectrum disorders | 54.3 (45.1 to 64.7) | -29.5 (-29.7 to -29.4) | 623.6 (517.1 to 742) | 1.2 (1 to 1.4) |  | 8.4 (5.5 to 12) | -29.4 (-31.8 to -27) | 96.5 (63 to 138.1) | 1.4 (-2 to 4.8) |
| Bipolar disorders | 73.6 (55.1 to 94.8) | -30.9 (-32.5 to -28.9) | 844.2 (632.5 to 1087.6) | -0.8 (-3.1 to 2.1) |  | 16.4 (9.2 to 25.4) | -30.9 (-33.9 to -27.6) | 187.8 (105.2 to 292) | -0.7 (-5 to 4) |
| Conduct disorder | 178.5 (128.2 to 238.3) | -25.6 (-26.9 to -24.5) | 2048 (1471.5 to 2735) | 6.9 (5 to 8.4) |  | 21.6 (12.2 to 34) | -25.5 (-27.6 to -23.4) | 248.4 (139.7 to 390.3) | 7 (4 to 10.1) |
| Depressive disorders | 269.5 (210.3 to 338.5) | -34.1 (-36.2 to -32.1) | 3093.3 (2413.8 to 3884.3) | -5.4 (-8.4 to -2.4) |  | 50 (31.9 to 74.6) | -34.1 (-36.2 to -32) | 574.4 (365.8 to 856.6) | -5.4 (-8.4 to -2.2) |
|  |  |  |  |  |  |  |  |  |  |
|  |  |  |  |  |  |  |  |  |  |
| Eating Disorders | 77.1 (54.4 to 108.4) | -35.8 (-38.9 to -32.3) | 885.3 (623.9 to 1244.5) | -7.7 (-12.3 to -2.7) |  | 16.5 (9.6 to 25.7) | -35.7 (-38.9 to -32.2) | 189.4 (109.6 to 295.4) | -7.7 (-12.2 to -2.7) |
| Idiopathic developmental intellectual disability | 41.3 (13.9 to 68.6) | -40.1 (-47.6 to -37.3) | 473.7 (159.1 to 787.1) | -14 (-24.8 to -9.9) |  | 2 (0.7 to 3.5) | -38.9 (-43.7 to -35.5) | 22.8 (8.2 to 40) | -12.2 (-19.1 to -7.3) |
| Schizophrenia | 7.9 (5.6 to 10.7) | -36.2 (-38.1 to -34.6) | 90.3 (64.8 to 122.4) | -8.4 (-11 to -6.1) |  | 5.2 (3.4 to 7.8) | -36.1 (-41.2 to -30.2) | 60.1 (39.6 to 90) | -8.2 (-15.5 to 0.3) |
| Other mental disorders | 46.2 (28.2 to 67.8) | -34.3 (-34.4 to -34.1) | 529.9 (323.4 to 778.4) | -5.6 (-5.7 to -5.4) |  | 3.6 (1.8 to 5.8) | -34.2 (-38.6 to -29.6) | 40.8 (20.8 to 67) | -5.5 (-11.7 to 1.1) |
