# Supplementary material for: Burden of mental health and substance use disorders among Italian young people aged 10–24 years: results from the Global Burden of Disease 2019 Study
Source: Soc Psychiatry Psychiatr Epidemiol. 2022 Jan 20;57(4):683–94. doi: 10.1007/s00127-022-02222-0 (PMC8960651; doi:10.1007/s00127-022-02222-0)
Supplement: Supplementary file 2 — Supplementary file2 (DOCX 21 KB) [file 127_2022_2222_MOESM2_ESM.docx]

**Online Resource 2**

Prevalence and years lived with disability (YLDs) for 2019, percentage change of prevalence and YLD counts, and percentage change of age-specific prevalence and YLD rates for 1990-2019 for both sexes combined for substance use disorders among young people aged 10-24 years in Italy, Western Europe and globally (Source: Global Burden of Disease study 2019; generated from data available at <http://ghdx.healthdata.org/gbd-results-tool>)

|  | **Prevalence** |  |  |  |  | **YLDs** |  |  |  |
| --- | --- | --- | --- | --- | --- | --- | --- | --- | --- |
|  | **2019 age-specific counts (thousands)** | **Percentage change in age specific counts, 1990-2019** | **2019 age -specific rate per 100,000 people** | **Percentage change**  **in age-specific**  **rates, 1990-2019** |  | **2019 age-specific counts (thousands)** | **Percentage change in age-specific counts, 1990-2019** | **2019 age -specific rate per 100,000 people** | **Percentage change**  **in age-specific**  **rates, 1990-2019** |
| **Globally** |  |  |  |  |  |  |  |  |  |
| *Substance use disorders* | 30780.3 (25068 to 37625.1) | 6.7 (3.6 to 9.8) | 1653.2 (1346.4 to 2020.8) | -11.3 (-13.8 to -8.6) |  | 4300 (2887.6 to 5923.4) | 12.7 (7.8 to 18.7) | 231 (155.1 to 318.1) | -6.3 (-10.3 to -1.3) |
| Alcohol use disorders | 13310.9 (8951.4 to 18718.7) | -4.1 (-6.8 to -1.6) | 714.9 (480.8 to 1005.4) | -20.2 (-22.4 to -18.1) |  | 1360.1 (803.7 to 2165.1) | -4.2 (-7.3 to -1.6) | 73.1 (43.2 to 116.3) | -20.3 (-22.9 to -18.1) |
| *Drug use disorders* | 18142 (14117.2 to 23326) | 16 (12 to 20.3) | 974.4 (758.2 to 1252.8) | -3.5 (-6.8 to 0.1) |  | 2939.9 (1913.1 to 4185.9) | 22.7 (15.7 to 29.8) | 157.9 (102.8 to 224.8) | 2.1 (-3.8 to 8) |
| Amphetamine use disorders | 2315.8 (1329.8 to 3374.1) | -19.1 (-23 to -16.2) | 124.4 (71.4 to 181.2) | -32.7 (-35.9 to -30.3) |  | 308.5 (154.8 to 520.5) | -19 (-24 to -15.3) | 16.6 (8.3 to 28) | -32.6 (-36.7 to -29.5) |
| Cannabis use disorders | 10689.5 (7066.2 to 16040.8) | 18 (13.3 to 23.5) | 574.1 (379.5 to 861.5) | -1.8 (-5.7 to 2.8) |  | 312.1 (169.6 to 519.7) | 18.1 (13.1 to 24.3) | 16.8 (9.1 to 27.9) | -1.7 (-5.9 to 3.4) |
| Cocaine use disorders | 1287.1 (885.1 to 1807.7) | 12 (6.7 to 18.2) | 69.1 (47.5 to 97.1) | -6.9 (-11.2 to -1.6) |  | 178.3 (101.5 to 285.6) | 11.7 (5.9 to 18.8) | 9.6 (5.5 to 15.3) | -7 (-11.9 to -1.2) |
| Opioid use disorders | 4267.5 (2969.9 to 6253.2) | 48.1 (41.3 to 58.4) | 229.2 (159.5 to 335.9) | 23.2 (17.6 to 31.8) |  | 1817 (1077.2 to 2842.1) | 47.4 (39.9 to 57.6) | 97.6 (57.9 to 152.6) | 22.6 (16.4 to 31.1) |
| Other drug use disorders | 70.2 (44.3 to 105) | 3 (-3.1 to 8.8) | 3.8 (2.4 to 5.6) | -14.3 (-19.4 to -9.5) |  | 324 (190.3 to 490.7) | -9.6 (-14.4 to -4.4) | 17.4 (10.2 to 26.4) | -24.8 (-28.8 to -20.5) |
|  |  |  |  |  |  |  |  |  |  |
| **Western Europe** |  |  |  |  |  |  |  |  |  |
| *Substance use disorders* | 2870.4 (2366.3 to 3496.6) | -20.2 (-23.6 to -16.9) | 4010 (3305.7 to 4884.8) | -8.4 (-12.3 to -4.6) |  | 326 (219.6 to 445.6) | -12.4 (-16.9 to -6.3) | 455.4 (306.8 to 622.5) | 0.6 (-4.6 to 7.5) |
| Alcohol use disorders | 1298.7 (833.8 to 1871.1) | -23.3 (-29.2 to -19.3) | 1814.3 (1164.8 to 2613.9) | -12 (-18.7 to -7.4) |  | 132.8 (77 to 217.1) | -23.4 (-29.3 to -18.8) | 185.5 (107.5 to 303.3) | -12 (-18.8 to -6.8) |
| *Drug use disorders* | 1644.6 (1375.8 to 1993.7) | -17.6 (-22.9 to -11.9) | 2297.6 (1922 to 2785.2) | -5.4 (-11.5 to 1.2) |  | 193.2 (130.4 to 266.7) | -2.8 (-9.7 to 5.9) | 269.8 (182.2 to 372.6) | 11.6 (3.7 to 21.6) |
| Amphetamine use disorders | 203 (119.4 to 294.5) | -19.3 (-22.6 to -15.8) | 283.5 (166.8 to 411.4) | -7.3 (-11.1 to -3.3) |  | 26.8 (13.4 to 45.7) | -19 (-25.3 to -11.9) | 37.4 (18.7 to 63.9) | -7.1 (-14.3 to 1.2) |
| Cannabis use disorders | 1096.7 (860.8 to 1432.8) | -24.1 (-29.5 to -18.5) | 1532.1 (1202.5 to 2001.6) | -12.9 (-19.1 to -6.5) |  | 32 (19.8 to 49) | -24.1 (-29.9 to -17.3) | 44.7 (27.6 to 68.4) | -12.9 (-19.6 to -5) |
| Cocaine use disorders | 199.6 (128.7 to 291.7) | 8.4 (2 to 15.3) | 278.8 (179.8 to 407.5) | 24.5 (17.1 to 32.3) |  | 27.6 (14.8 to 45.9) | 8.4 (-2.1 to 20.2) | 38.5 (20.7 to 64.1) | 24.4 (12.4 to 38) |
| Opioid use disorders | 165.9 (116.2 to 236.3) | 19.7 (6.6 to 33) | 231.8 (162.3 to 330) | 37.4 (22.3 to 52.6) |  | 70.6 (42.3 to 108.1) | 19.7 (5.3 to 35.2) | 98.6 (59.1 to 151) | 37.4 (20.9 to 55.2) |
| Other drug use disorders | 9.2 (6.2 to 13) | -11 (-16.4 to -4.3) | 12.8 (8.6 to 18.2) | 2.2 (-4.1 to 9.8) |  | 36.3 (21.8 to 54.8) | -7.2 (-16 to 3.5) | 50.7 (30.4 to 76.5) | 6.5 (-3.6 to 18.9) |
|  |  |  |  |  |  |  |  |  |  |
| **Italy** |  |  |  |  |  |  |  |  |  |
| *Substance use disorders* | 278.4 (216.8 to 354.8) | -47.6 (-51.4 to -44.1) | 3195.4 (2487.5 to 4072.2) | -24.7 (-30.1 to -19.6) |  | 29.8 (19.8 to 41.8) | -47.3 (-51.1 to -43.9) | 341.5 (227.1 to 480) | -24.2 (-29.8 to -19.3) |
| Alcohol use disorders | 132.5 (90.1 to 184.7) | -33.3 (-35.4 to -31) | 1520.4 (1034.5 to 2120.1) | -4.1 (-7.1 to -0.9) |  | 13.6 (8.1 to 21.5) | -33.2 (-36.3 to -29.7) | 155.8 (92.5 to 247.2) | -4 (-8.4 to 1) |
| *Drug use disorders* | 151.4 (106.5 to 218.3) | -56 (-59.7 to -52) | 1736.9 (1221.8 to 2505.2) | -36.7 (-42.1 to -31.1) |  | 16.2 (10.3 to 23.4) | -55.2 (-59.5 to -51.2) | 185.6 (118.3 to 268.1) | -35.6 (-41.9 to -29.9) |
| Amphetamine use disorders | 27.8 (15.6 to 41.7) | -46.2 (-48.6 to -43.4) | 319.6 (178.7 to 478.2) | -22.8 (-26.2 to -18.7) |  | 3.7 (1.8 to 6.4) | -46.1 (-51 to -41.1) | 42.2 (20.3 to 73.7) | -22.6 (-29.6 to -15.4) |
| Cannabis use disorders | 102.8 (61.1 to 167.3) | -58.5 (-63.7 to -53.2) | 1180.1 (701.2 to 1920) | -40.4 (-47.8 to -32.7) |  | 3 (1.5 to 5.5) | -58.5 (-63.8 to -52.4) | 34.4 (16.7 to 63.5) | -40.4 (-48.1 to -31.6) |
| Cocaine use disorders | 12.1 (7.4 to 18.3) | -27.7 (-30.8 to -23.7) | 139.3 (84.6 to 210.5) | 3.9 (-0.6 to 9.7) |  | 1.7 (0.8 to 2.9) | -27.6 (-36.9 to -17.3) | 19.3 (9.7 to 33) | 4 (-9.3 to 18.8) |
| Opioid use disorders | 10 (6.4 to 15.2) | -68.8 (-74.9 to -62.8) | 115 (73.4 to 174.7) | -55.1 (-63.9 to -46.5) |  | 4.3 (2.4 to 7) | -68.8 (-75.3 to -62.1) | 49 (27.5 to 80.9) | -55.2 (-64.5 to -45.6) |
| Other drug use disorders | 0.6 (0.4 to 0.9) | -31.1 (-35 to -26.8) | 7.1 (4.3 to 10.8) | -1 (-6.6 to 5.2) |  | 3.5 (2 to 5.7) | -41.3 (-47.6 to -33.6) | 40.7 (22.8 to 65.6) | -15.6 (-24.8 to -4.6) |
