# Supplementary material for: Burden of mental health and substance use disorders among Italian young people aged 10–24 years: results from the Global Burden of Disease 2019 Study
Source: Soc Psychiatry Psychiatr Epidemiol. 2022 Jan 20;57(4):683–94. doi: 10.1007/s00127-022-02222-0 (PMC8960651; doi:10.1007/s00127-022-02222-0)

**Online Resource 3**

Figure that illustrates trends in YLDs rates (per 100,000 young people aged 10-24) due to **a** anxiety disorders (AD) **b** attention-deficit/hyperactivity disorder (ADHD) **c** autism spectrum disorders (ASD) **d** bipolar disorders (BD) **e** conduct disorder (CD) **f** depressive disorders (DD) **g** eating disorders (ED) **h** idiopathic developmental intellectual disability (IDID) **i** schizophrenia (SZ) and **l** other mental disorders (OMD) by sex (Male: blue dotted line; Female: solid red line)

**a**


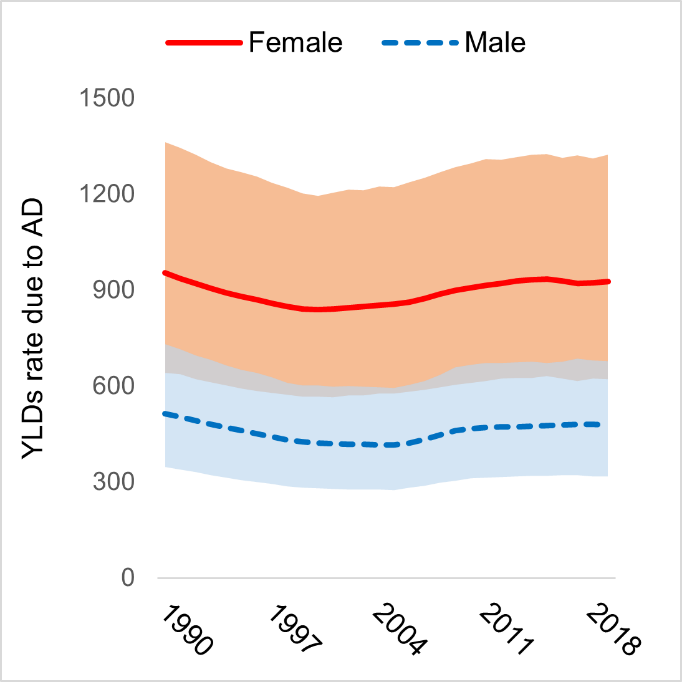


**b**


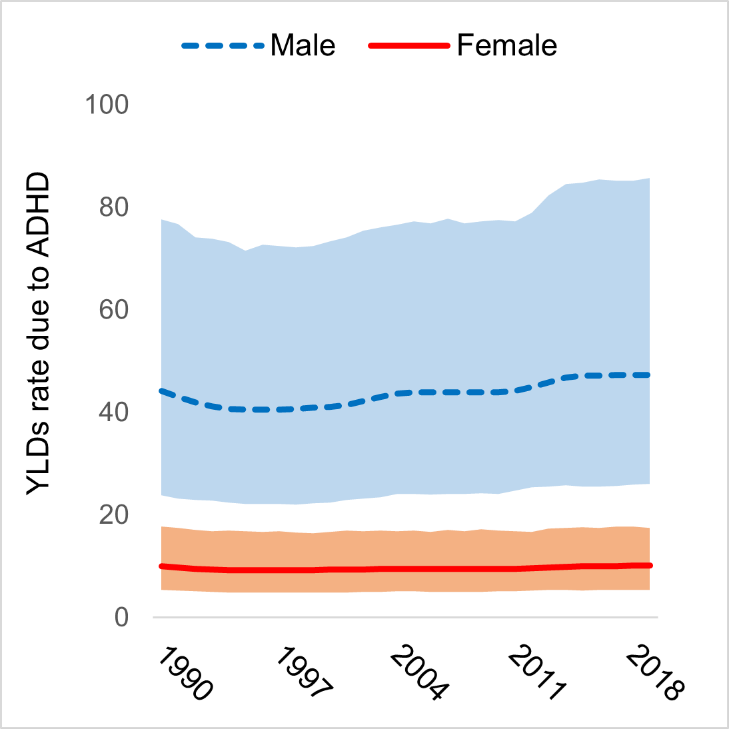


**c**


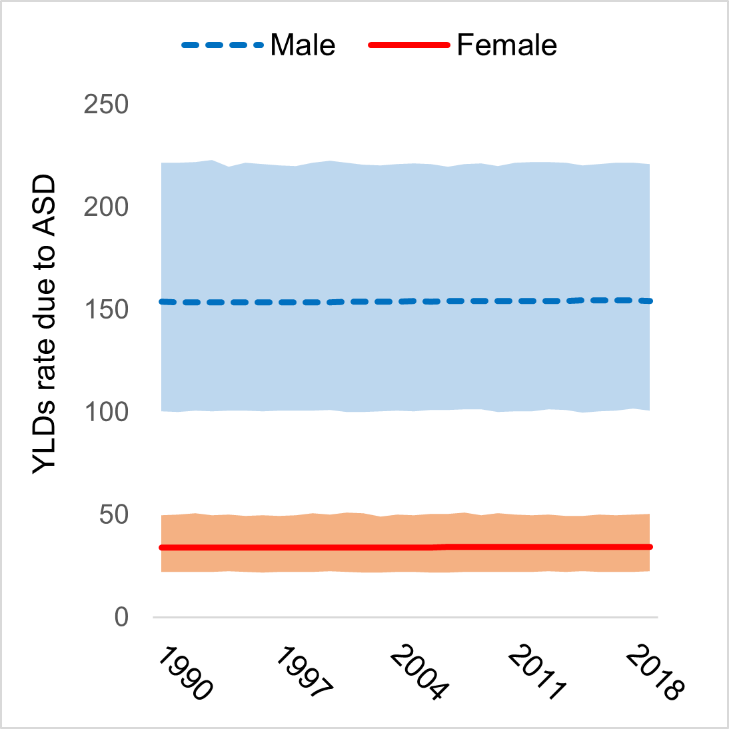


**d**


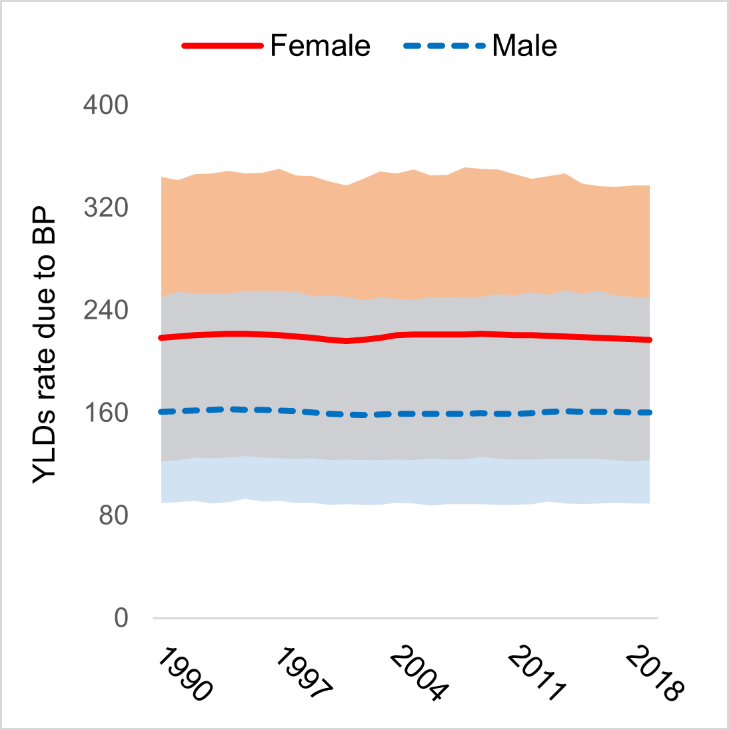


**e**


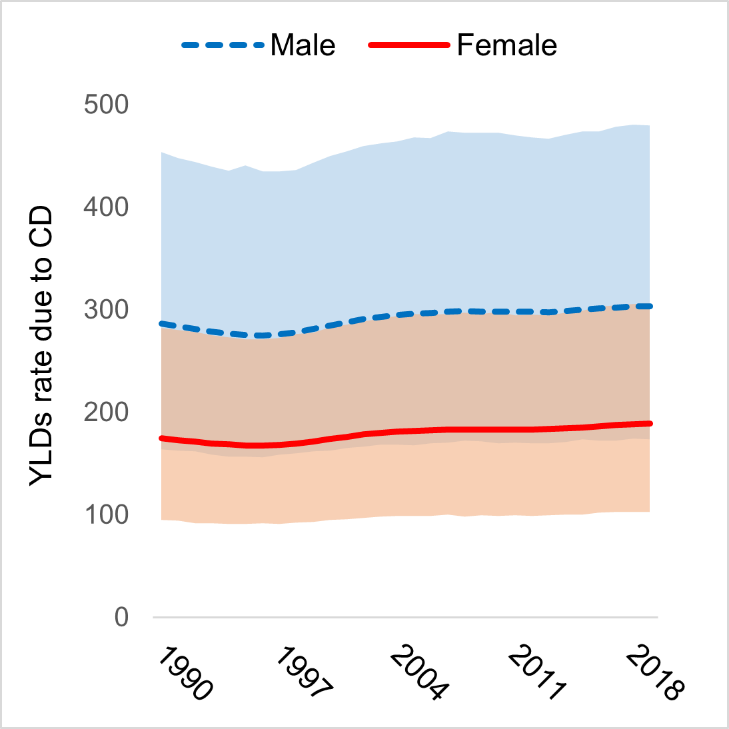


**f**


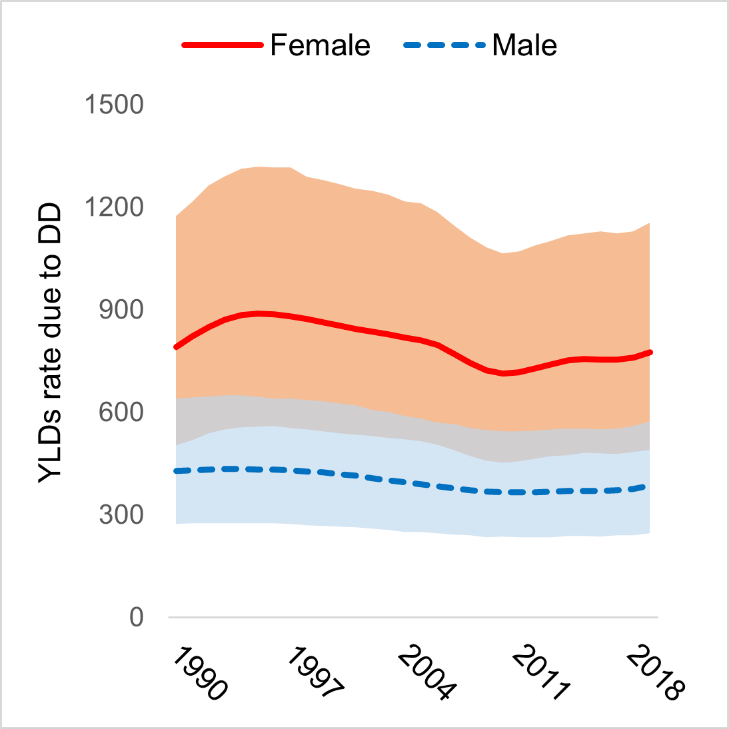


**g**


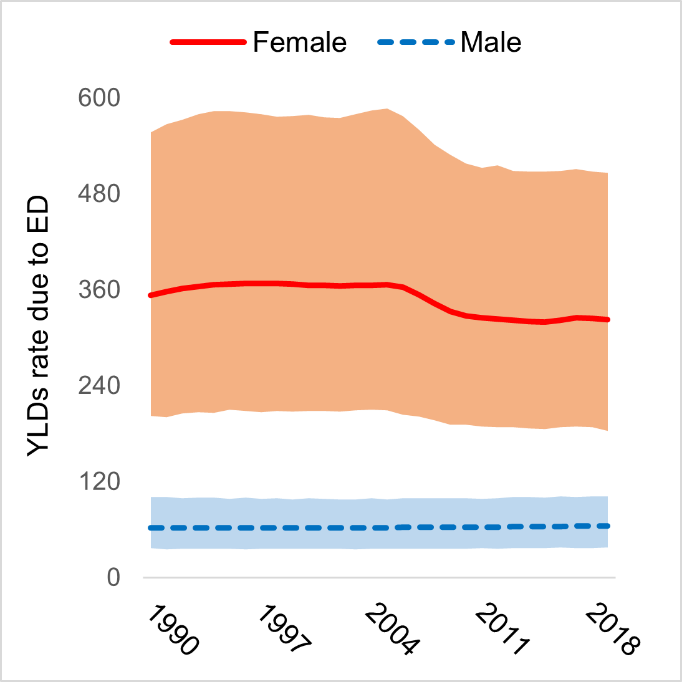


**h**


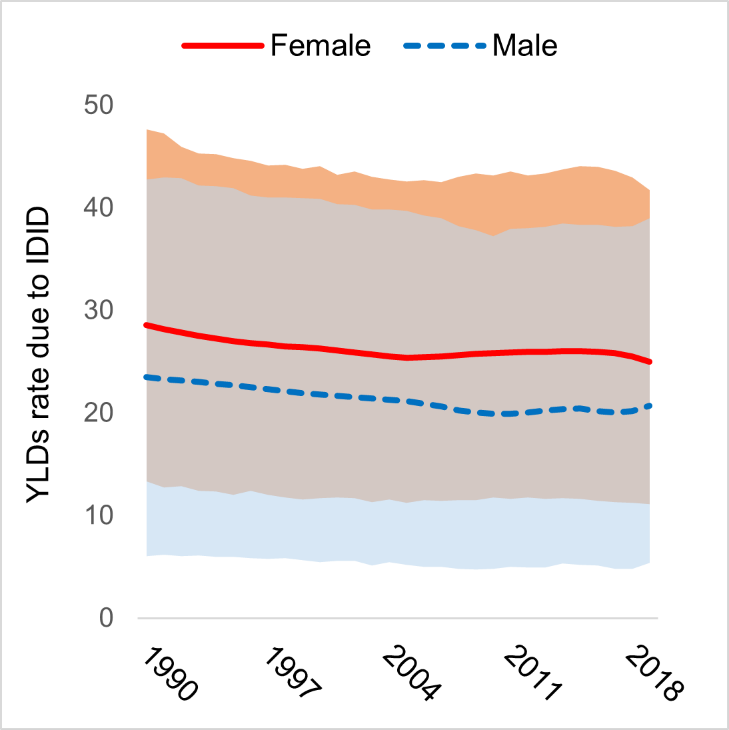


**i**


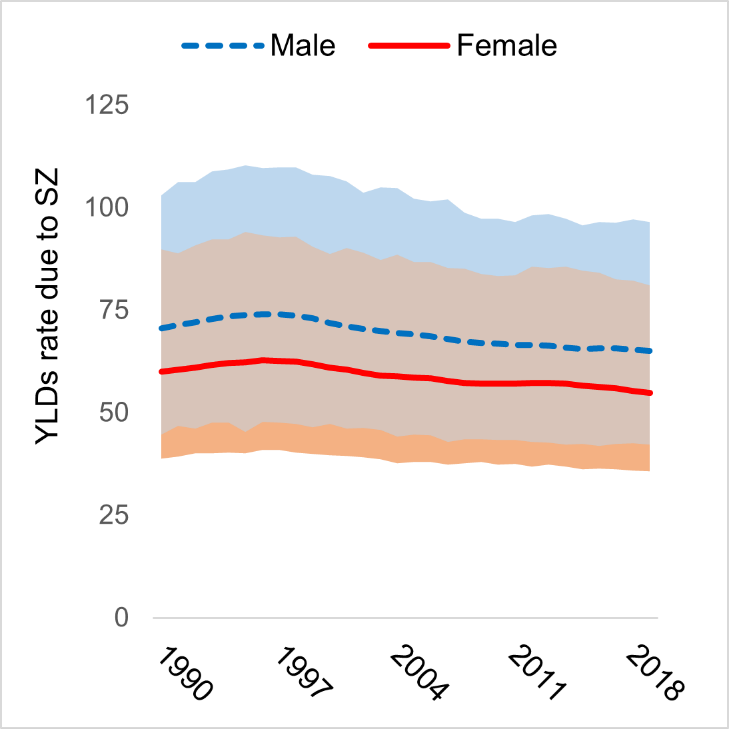


**l**


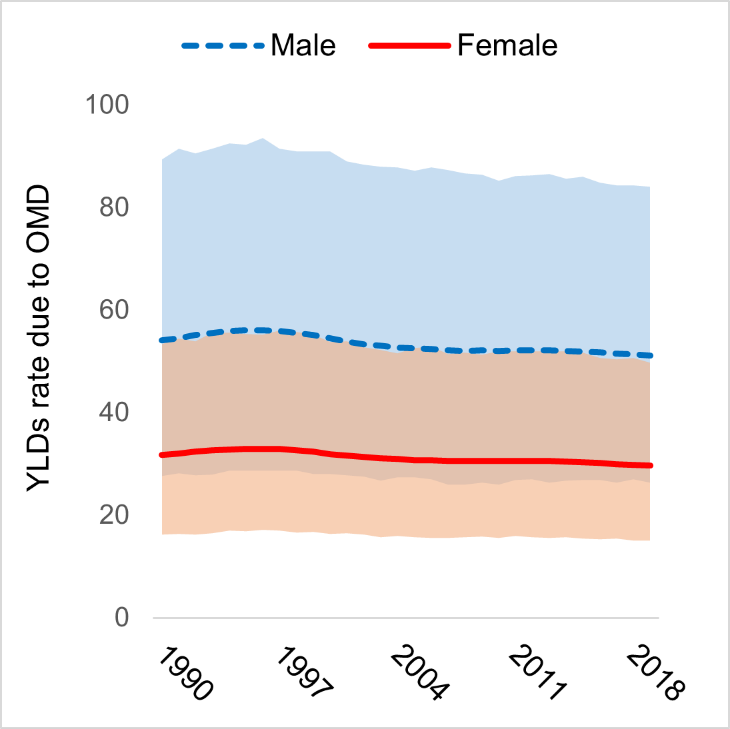

Supplement: Supplementary file 3 — Supplementary file3 (DOCX 880 KB) [file 127_2022_2222_MOESM3_ESM.docx]
