# Supplementary material for: Burden of mental health and substance use disorders among Italian young people aged 10–24 years: results from the Global Burden of Disease 2019 Study
Source: Soc Psychiatry Psychiatr Epidemiol. 2022 Jan 20;57(4):683–94. doi: 10.1007/s00127-022-02222-0 (PMC8960651; doi:10.1007/s00127-022-02222-0)
Supplement: Supplementary file 4 — Supplementary file4 (DOCX 601 KB) [file 127_2022_2222_MOESM4_ESM.docx]

**Online Resource 4**

Figure that illustrates trends in YLDs rates (per 100,000 young people aged 10-24) due to **a** alcohol use disorders (AUD) **b** amphetamine use disorder (AMUD) **c** cannabis use disorders (CAUD) **d** cocaine use disorders (CUD) **e** opioid use disorders (OUD) and **f** other drug use disorders (ODUD) by sex (Male: blue dotted line; Female: solid red line)

**a**

**
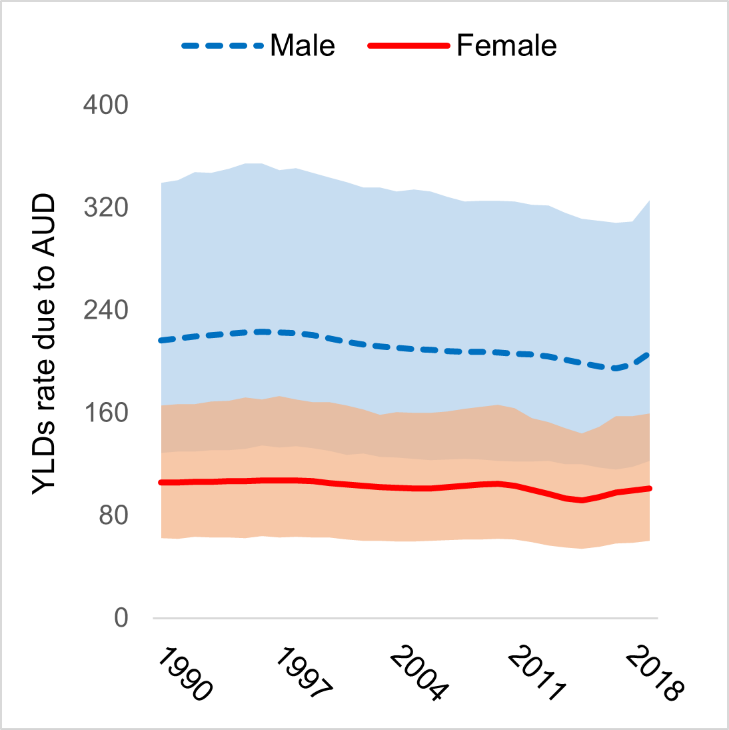
**

**b**

**
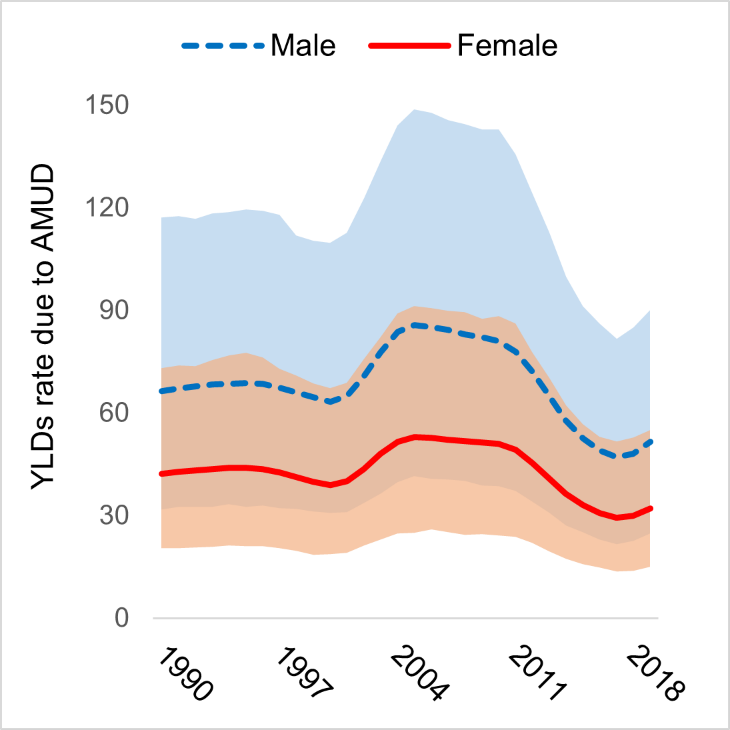
**

**c**

**
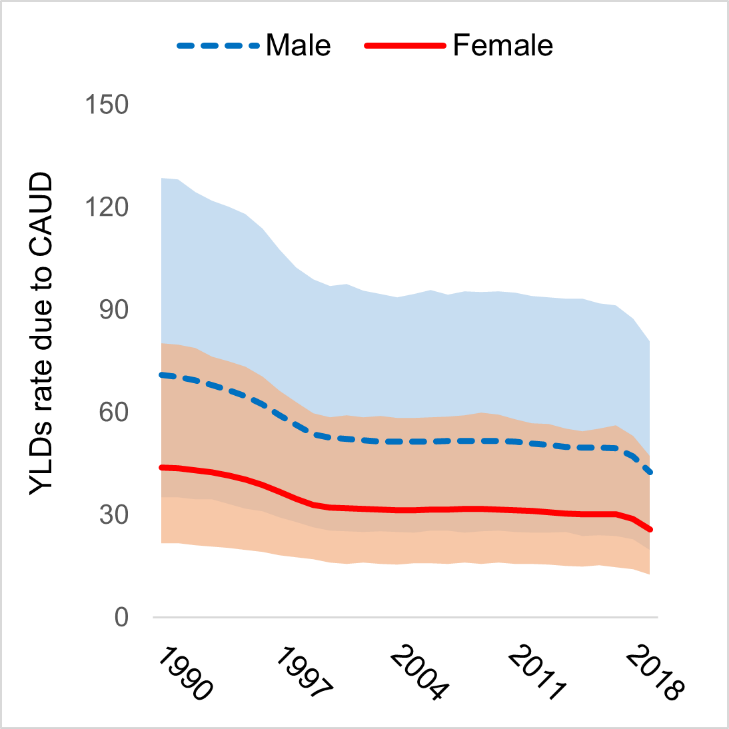
**

**d**

**
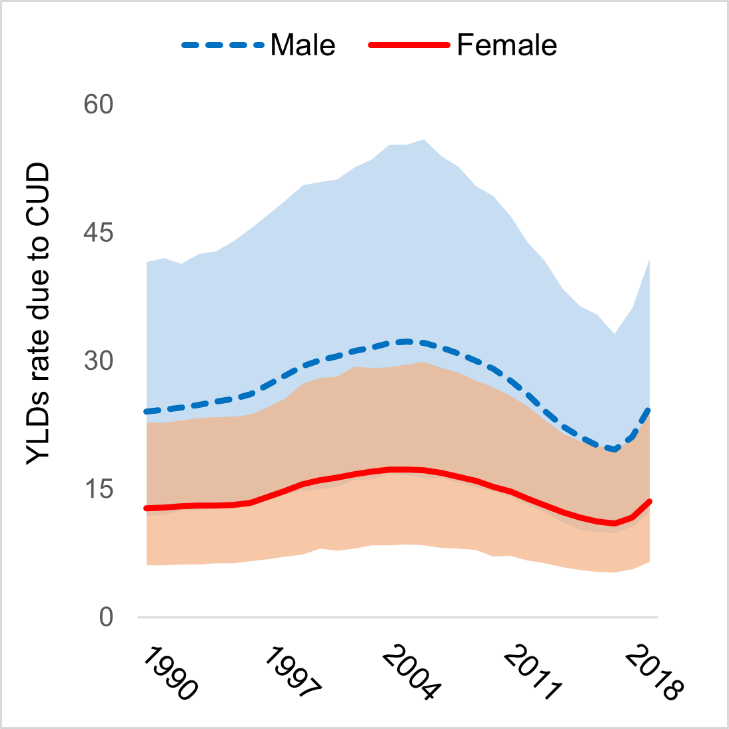
**

**e**

**
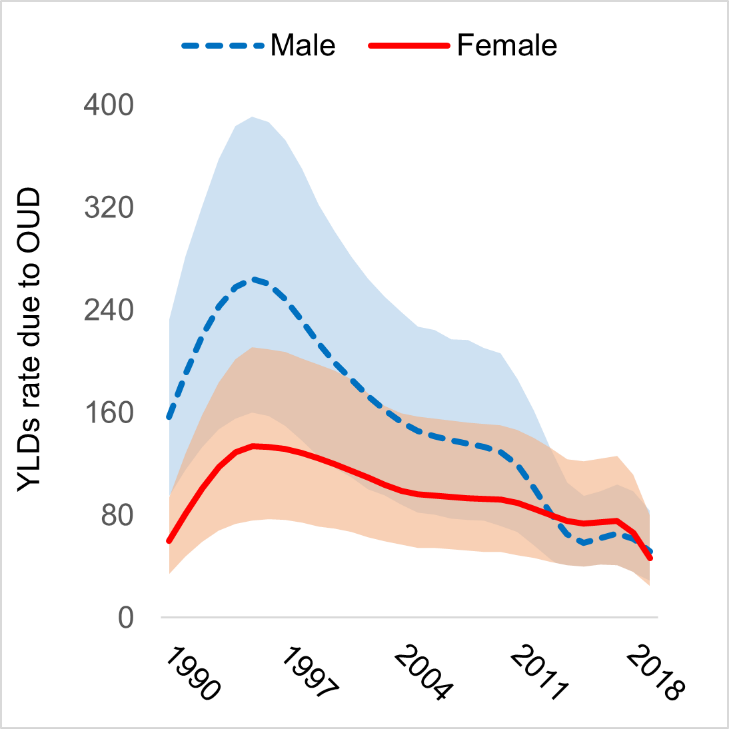
**

**f**

**
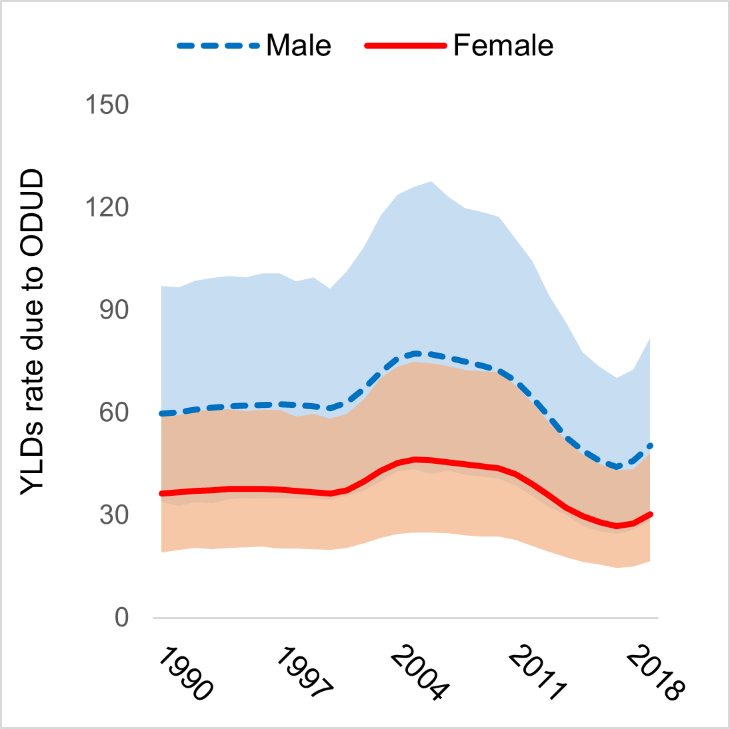
**
