# Supplementary material for: Burden of mental health and substance use disorders among Italian young people aged 10–24 years: results from the Global Burden of Disease 2019 Study
Source: Soc Psychiatry Psychiatr Epidemiol. 2022 Jan 20;57(4):683–94. doi: 10.1007/s00127-022-02222-0 (PMC8960651; doi:10.1007/s00127-022-02222-0)
Supplement: Supplementary file 5 — Supplementary file5 (DOCX 20 KB) [file 127_2022_2222_MOESM5_ESM.docx]

**Online Resource 5**

Prevalence and years lived with disability (YLDs) rates (per 100,000) by sex and age-group for mental disorders among Italian young people in 2019 (Source: Global Burden of Disease study 2019; generated from data available at <http://ghdx.healthdata.org/gbd-results-tool>)

|  | **Sex** | **Age -specific prevalence rate per 100,000 people** |  |  |  | **Age -specific YLDs rate per 100,000 people** |  |  |
| --- | --- | --- | --- | --- | --- | --- | --- | --- |
|  |  | **10 – 14 years** | **15 – 19 years** | **20 – 24 years** |  | **10 – 14 years** | **15 – 19 years** | **20 – 24 years** |
| *Mental disorders* | M | 15808.3 (13471.8 to 18670.9) | 16339.9 (14402.7 to 18568.5) | 13825.7 (12142.6 to 15878.1) |  | 1509.7 (1038.1 to 2137) | 1891.3 (1317.2 to 2589.4) | 1787.8 (1269.2 to 2408.1) |
|  | F | 14903 (12542.1 to 17775.5) | 19446.1 (16960.3 to 22231.6) | 19812.8 (17110.2 to 22757.8) |  | 1832.8 (1248 to 2575.3) | 2829.9 (1944.7 to 3897.3) | 3092 (2163.1 to 4221.4) |
| Anxiety disorders | M | 4266.5 (3050.7 to 5753.3) | 5173.3 (3998.6 to 6649.3) | 4980.9 (3680.6 to 6585.2) |  | 427.6 (268 to 633.9) | 512.8 (337.4 to 735.1) | 492.5 (315.8 to 729.5) |
|  | F | 8407.6 (6026.2 to 11200.2) | 10165.3 (7859.7 to 12919.7) | 9794.2 (7331.4 to 12942.9) |  | 837.9 (532.8 to 1241.3) | 994.8 (669 to 1415.9) | 948.4 (614.8 to 1398.8) |
| Attention-deficit/hyperactivity disorder | M | 5380.5 (3526.1 to 7900.5) | 3878.1 (2636.4 to 5658.6) | 2421.9 (1673 to 3627.2) |  | 66.1 (34.4 to 120.3) | 47.3 (25.2 to 86.2) | 29.5 (15.8 to 52.3) |
|  | F | 1099.3 (711.4 to 1591) | 842.7 (559.6 to 1205.6) | 567.1 (371.6 to 826.6) |  | 13.4 (7.1 to 23.9) | 10.2 (5.4 to 17.8) | 6.8 (3.5 to 12.4) |
| Autism spectrum disorders | M | 1023.5 (849.9 to 1212.5) | 996.6 (832 to 1180.6) | 969.1 (805.4 to 1147.4) |  | 159.8 (103.7 to 228.4) | 154.1 (100.8 to 222) | 149.8 (97.2 to 216) |
|  | F | 229.7 (185.4 to 278.2) | 223.9 (181.6 to 271.2) | 218.2 (177.9 to 264.6) |  | 35.6 (23.2 to 52.7) | 34.3 (21.9 to 50.8) | 33.1 (21.7 to 48.4) |
| Bipolar disorders | M | 232.9 (159 to 324.6) | 855.7 (607.2 to 1154.8) | 1038.2 (783.8 to 1323.3) |  | 53.1 (27 to 86.7) | 192.2 (104.5 to 304) | 232.6 (131.3 to 364.7) |
|  | F | 302.1 (204.8 to 420.9) | 1144.9 (825.7 to 1519.7) | 1495.4 (1134.3 to 1897.1) |  | 68.3 (35.2 to 113.6) | 253.8 (138 to 405.5) | 327.5 (186.3 to 517.8) |
| Conduct disorder | M | 4488.3 (3274.2 to 5954.5) | 3085.9 (2181 to 4214.9) | - - |  | 549.2 (308.8 to 891.8) | 373.3 (206.2 to 593.6) | - - |
|  | F | 2989.7 (2023.6 to 4200.5) | 1754.1 (1135.6 to 2609.3) | - - |  | 362.7 (190.7 to 611.6) | 208.7 (107.9 to 339.7) | - - |
| Depressive disorders | M | 1003.3 (689.2 to 1381.2) | 2338.2 (1725.8 to 3028) | 2972 (2205.3 to 3868.5) |  | 192.8 (109.4 to 316.2) | 434.3 (268.7 to 666.6) | 523.4 (320.1 to 789.3) |
|  | F | 1876.4 (1269.5 to 2617.1) | 4632.1 (3484.5 to 5935.6) | 5882.6 (4308.6 to 7688.9) |  | 365 (207.6 to 606.7) | 880 (546 to 1339.9) | 1080.2 (658 to 1652.7) |
| Eating Disorders | M | 132.8 (81.6 to 207.9) | 347.1 (222.3 to 549.3) | 411.8 (236.6 to 621.4) |  | 28.9 (15.1 to 49.3) | 75.1 (39.3 to 129.9) | 88.8 (46.6 to 153.3) |
|  | F | 534.5 (312.7 to 897.8) | 1697.4 (1083.6 to 2727.1) | 2297.7 (1500.3 to 3224.6) |  | 115.6 (57.4 to 204.3) | 363.3 (188.3 to 638) | 488.2 (275.9 to 781.7) |
| Idiopathic developmental intellectual disability | M | 434.3 (85.4 to 786.1) | 430.7 (87.6 to 776.4) | 424.8 (89.8 to 762.8) |  | 20.8 (5.4 to 39.1) | 20.7 (5.3 to 39.4) | 20.7 (5.5 to 38.7) |
|  | F | 531.2 (234.1 to 826.7) | 520.8 (226.5 to 813.3) | 510.7 (221.3 to 799) |  | 25.7 (11.3 to 43.8) | 24.9 (10.9 to 41.5) | 24.4 (10.6 to 40.6) |
| Schizophrenia | M | 7 (3.6 to 11.7) | 59.5 (40.2 to 85.3) | 218.8 (153.1 to 303.2) |  | 5 (2.5 to 8.4) | 39.9 (23 to 61.3) | 147.1 (92.9 to 219.5) |
|  | F | 6.6 (3.4 to 10.8) | 53.8 (35.7 to 76.9) | 188.2 (130.5 to 262) |  | 4.7 (2.3 to 7.8) | 35.6 (21.2 to 54.3) | 123.1 (77.7 to 185.9) |
| Other mental disorders | M | 79.3 (47 to 117.8) | 539.4 (320.2 to 801.8) | 1337.9 (837.2 to 1932.2) |  | 6.3 (3.1 to 10.6) | 41.7 (21.1 to 69.3) | 103.3 (54.3 to 172.6) |
|  | F | 46.6 (25.7 to 72.2) | 317.5 (174.6 to 491.3) | 795.3 (462.9 to 1208.1) |  | 3.7 (1.8 to 6.5) | 24.4 (11.6 to 42) | 60.3 (31.4 to 101.5) |
