# Supplementary material for: Burden of mental health and substance use disorders among Italian young people aged 10–24 years: results from the Global Burden of Disease 2019 Study
Source: Soc Psychiatry Psychiatr Epidemiol. 2022 Jan 20;57(4):683–94. doi: 10.1007/s00127-022-02222-0 (PMC8960651; doi:10.1007/s00127-022-02222-0)
Supplement: Supplementary file 6 — Supplementary file6 (DOCX 18 KB) [file 127_2022_2222_MOESM6_ESM.docx]

**Online Resource 6**

Prevalence and years lived with disability (YLDs) rates (per 100,000) by sex and age-group substance use disorders among Italian young people in 2019 (Source: Global Burden of Disease study 2019; generated from data available at <http://ghdx.healthdata.org/gbd-results-tool>)

|  | **Sex** | **Age -specific prevalence rate per 100,000 people** |  |  |  | **Age -specific YLDs rate per 100,000 people** |  |  |
| --- | --- | --- | --- | --- | --- | --- | --- | --- |
|  |  | **10 – 14 years** | **15 – 19 years** | **20 – 24 years** |  | **10 – 14 years** | **15 – 19 years** | **20 – 24 years** |
| *Substance use disorders* | M | 280.8 (138 to 490.9) | 4038.2 (2773.2 to 5763.7) | 7613.1 (5972.3 to 9591.3) |  | 17.5 (9.5 to 29.3) | 341.5 (222.1 to 491.7) | 904 (600.1 to 1287.9) |
|  | F | 194.1 (113.2 to 308.2) | 2489.7 (1749.8 to 3527.1) | 4187.1 (3289 to 5245.9) |  | 13.3 (7.5 to 21.8) | 208 (136.6 to 296.5) | 521.5 (347.8 to 727.3) |
| Alcohol use disorders | M | 96.6 (55.6 to 158.7) | 1497.6 (988.6 to 2098.8) | 4334.1 (2872.7 to 6094.1) |  | 10.2 (5.1 to 18.5) | 154.4 (86.9 to 244.8) | 446.1 (260 to 708.9) |
|  | F | 80.9 (46 to 131.7) | 838.8 (565.4 to 1157.5) | 2054.9 (1310.3 to 2928.2) |  | 8.5 (4.3 to 15.2) | 85.4 (48.5 to 134.2) | 207.7 (119.9 to 338.1) |
| *Drug use disorders* | M | 196.8 (57.4 to 399.6) | 2587.8 (1419.9 to 4238.8) | 3490.8 (2525.2 to 4752.8) |  | 7.3 (2.7 to 14.8) | 187.1 (115 to 280.8) | 457.9 (288.6 to 663) |
|  | F | 124.1 (53.9 to 229.5) | 1668.1 (1019.4 to 2667.8) | 2192 (1601.6 to 2900.2) |  | 4.8 (2.1 to 9) | 122.6 (75.3 to 185.2) | 313.9 (196.8 to 456.9) |
| Amphetamine use disorders | M | - - | 199.7 (108.5 to 301.5) | 942.7 (528.4 to 1411.4) |  | - - | 26.7 (12.6 to 47.7) | 125.4 (59.5 to 220.1) |
|  | F | - - | 128.4 (69.1 to 195.3) | 603.8 (334.4 to 906.8) |  | - - | 16.9 (7.4 to 29.8) | 78.6 (36.9 to 136.2) |
| Cannabis use disorders | M | 196.8 (55.4 to 397.2) | 2119.8 (955.9 to 3824.7) | 2006.6 (1184.5 to 3205.6) |  | 5.8 (1.5 to 13.2) | 62.2 (25.2 to 125.1) | 58.7 (28.5 to 104.8) |
|  | F | 132 (56.2 to 245.7) | 1366.5 (721.2 to 2382.5) | 1163 (680.3 to 1817) |  | 3.9 (1.4 to 8) | 39.6 (17.6 to 77.3) | 33.5 (16.5 to 59.6) |
| Cocaine use disorders | M | 6.2 (2.8 to 10.7) | 237.6 (128.8 to 385.8) | 280.6 (177.4 to 413.1) |  | 0.9 (0.4 to 1.7) | 33.2 (15.2 to 60.4) | 39 (19.9 to 68) |
|  | F | 3.5 (1.5 to 6.4) | 135.1 (70.6 to 228.6) | 158.6 (97.6 to 240.9) |  | 0.5 (0.2 to 1) | 18.7 (7.5 to 36.1) | 21.5 (10.5 to 37.9) |
| Opioid use disorders | M | - - | 60.2 (37.6 to 92.7) | 291.8 (190.9 to 433.5) |  | - - | 26 (13.3 to 44.2) | 125.6 (70.4 to 203.4) |
|  | F | - - | 56.5 (32.8 to 93.3) | 271.1 (167.5 to 433.4) |  | - - | 24.2 (11.3 to 43.1) | 113.7 (62 to 195.6) |
| Other drug use disorders | M | 0 (0 to 0) | 1.9 (0.9 to 3.8) | 22.8 (14.4 to 34.4) |  | 0.6 (0.2 to 1.1) | 39 (20.9 to 64.4) | 109.2 (61 to 177.8) |
|  | F | 0 (0 to 0) | 1.4 (0.6 to 2.9) | 15.6 (8.8 to 24.8) |  | 0.3 (0.1 to 0.7) | 23.2 (11.9 to 37.7) | 66.6 (36.3 to 107.8) |
